# Transcriptional Response of *Candida albicans* to Nanostructured Surfaces Provides Insight into Cellular Rupture and Antifungal Drug Sensitization

**DOI:** 10.1021/acsbiomaterials.3c00938

**Published:** 2023-11-17

**Authors:** Lakshmi Gayitri Chivukula, Dennis LaJeunesse

**Affiliations:** Department of Nanoscience, Joint School of Nanoscience and Nanoengineering, University of North Carolina Greensboro, 2907 East Lee Street, Greensboro, North Carolina 27455, United States

**Keywords:** nanoscale antimicrobial materials, nanostructured surfaces, *Candida albicans*, gene expression, ergosterol biosynthesis, antifungal drug resistance, biofilm

## Abstract

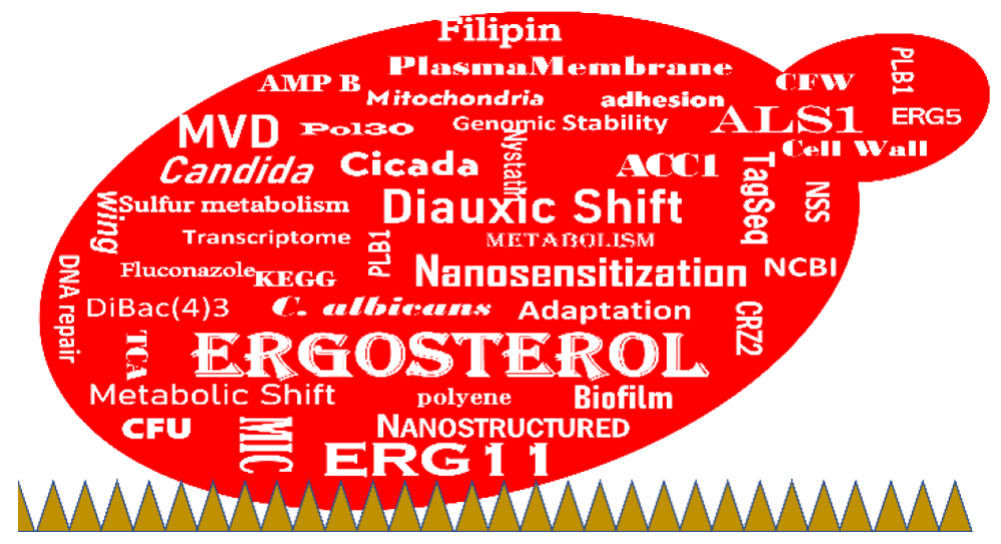

The rise in resistance levels against antifungal drugs has necessitated the development of strategies to combat fungal infections. Nanoscale antimicrobial surfaces, found on the cuticles of insects, have recently emerged as intriguing alternative antifungal strategies that function passively via contact and induced cell rupture. Nanostructured surfaces (NSS) offer a potentially transformative antimicrobial approach to reducing microbial biofilm formation. We examined the transcriptional response of *Candida albicans*, an opportunistic pathogen that is also a commensal dimorphic fungus, to the NSS found in the wings of *Neotibicen* spp. cicada and found characteristic changes in the expression of *C. albicans* genes associated with metabolism, biofilm formation, ergosterol biosynthesis, and DNA damage response after 2 h of exposure to the NSS. Further validation revealed that these transcriptional changes, particularly in the ergosterol biosynthesis pathway, sensitize *C. albicans* to major classes of antifungal drugs. These findings provide insights into NSS as antimicrobial surfaces and as a means of controlling biofilm formation.

## Introduction

The utilization of drug-based treatments for fungal infections has resulted in a rise in resistance levels against antifungal drugs, necessitating the development of novel strategies to combat them.^1,2^ Recently alternative antifungal strategies have been proposed, and among these are nanoscale antimicrobial materials, many of which can be found on the cuticles of insects, that are intriguing in that they function passively via contact and induced cell rupture due to an incompatibility between their surface structure and the cell itself.^2,3^ Nanostructured surfaces (NSS) offer a potentially groundbreaking approach to combating microbial biofilm formation. This is achieved through the antimicrobial effect generated by the physical interplay between the surface and cell, potentially circumventing certain adaptive mechanisms that typically lead to drug resistance. Both prokaryotic and eukaryotic microbes rupture on natural and synthetic NSS.^3−5^ Bacteria, particularly Gram-negative bacteria, rupture at high rate and efficiency,^6,7^ while eukaryotic microbes, particularly yeasts such as *Saccharomyces cerevisiae* and *Candida albicans*, exhibit less dramatic cellular rupture.^8,9^ The interactions of NSS from *Neotibicen* spp. cicada with *C. albicans* and *S. cerevisiae* also result in changes to adhesion, morphology, and metabolism, which suggests that yeast cells may also adapt to these surfaces.^9^ Difference between prokaryotic and eukaryotic microbial responses to NSS has been attributed to differences in the structures and organization of the cell walls as well as differences in adhesion mechanisms.^4^ We hypothesized that cellular fungi have adaptive mechanisms to respond to such nanoscale mechanical challenges. Here we examine the transcriptional response of *C. albicans* to an NSS that is found in the wings of the annual dog day/*Neotibicen* spp. cicada. Following a 2 h exposure to a nanostructured surface (NSS), discernible alterations occur in the expression of genes related to metabolism, biofilm formation, ergosterol biosynthesis, and DNA damage response in *C. albicans*. Validation of these results demonstrated that transcriptional changes in the ergosterol biosynthesis pathway result in sensitization of *C. albicans* to several major classes of antifungal drugs. These findings provide a potential molecular approach to design efficient antimicrobial surfaces to control biofilm formation in immunocompromised patients.

## Materials and Methods

### Yeast Culture Conditions

*Candida albicans* wild type (ATCC 90028) strain was used; these cells were cultured in Sabouraud Dextrose Broth (SDB) at 25 °C. Cell density was calculated by a colony forming unit (CFU) assay.^9^*C. albicans* was cultured under one of four conditions for these experiments: (1) on an NSS (experimental), (2) a glass coverslip (negative control), (3) a glass coverslip treated with PEG3000 (reduced adhesion control), and (4) cell passed through a 1 mm silicon tube (mechanical shear control). In each case, 10^6^ cells were used, corresponding roughly to 1 mL of cells grown to an OD600 of 0.2. For RNA extractions, *C. albicans* cells were incubated in SBD without shaking on the surfaces, i.e., NSS, the glass, and the poly(ethylene glycol) (PEG) treated glass for 2 h. In the case of the mechanical sheared control, cells were pushed through 2 m of 1 mm of silicon tubing and then incubated on a flat glass surface for 2 h. The NSS were from the wing of the cicada, and *Neotibicen* spp. were purchased at BioQuip Products, Inc., California. The preparation of the wings using these experiments was as follows: Wings from whole cicadas were carefully dissected from the organism. Isolated wings were sonicated in 70% ethanol for 10 min to remove any contaminants and air-dried at room temperature. Glass coverslips were cleaned via sonication in 70% ethanol for 10 min.

### Plasma Membrane

To measure perturbation to the plasma membrane, we examined changes to potential of the plasma membrane using the vital dye DiBAC4(3) (ex490/em516, Thermofisher B438); living *C. albicans* cells were labeled with 1 μM DiBAC4(3) for 10 min. In these experiments, yeast cells were also labeled with 5 μM calcofluor white (CFW) dye (ex380/em478; Thermofisher R40015) to examine chitin. Confocal micrographs were taken using a Zeiss Axio spinning disc confocal microscope using the 63× objective, and samples were excited using the 405 nm laser line to capture CFW fluorescence and the 488 nm laser line to capture DiBac4(3) fluorescence. *C. albicans* cells were cultured as described above on an NSS or flat glass surfaces. In these experiments, confocal micrographs were collected at four different time points: 0, 1, 2, and 4 h. All the experiments were performed in triplicate and evaluated separately.

### Differential Gene Expression/TagSeq Analysis

RNA was extracted from these cells via a RiboPure yeast RNA extraction kit. Taq sequencing was performed at The Genomic Sequencing and Analysis Facility, University of Texas, Austin (https://research.utexas.edu/cbrs/cores/genomics/). The Tag-Seq was done using a NovaSeq 6000 SR100 with standard coverage. The raw sequencing read quality of transcriptome was verified by FastQC v0.11.8. The low-quality reads and ambiguous reads were removed using trimmomatic flexible trimming tool v0.38.0 before mapping. The filtered/trimmed reads were mapped to the *Candida albicans* reference genome SC5314 (GCA_000182965.3). The read mapping was done using HISAT2 v2.1.0, which uses Bowtie2 as core processor. We assembled Tag-Seq read alignments into transcripts using StringTie v2.1.1. The quantification of differentially expressed genes between NSS response and flat surface (control) annotated to reference genome was done using DESeq2 v2.11.40.6. The DEGs with log2FC cutoff >0.8 and statistical significance (*P* < 0.01) was considered for the comparison. We used galaxy.org for transcriptome data analysis.^10^

### Gene Enrichment Analysis

Analyzing the gene enrichment and functional annotation of differentially expressed genes was done using Database for Annotation, Visualization and Integrated Discovery (DAVID) Bioinformatics tool v6.8.^11^ Identification and categorization of the enriched cellular and metabolic processes were performed using gene ontology enrichment analysis. Besides, detection of significantly enriched pathways in which DEGs were involved was searched using the Kyoto Encyclopedia of Genes and Genomes (KEGG) database.^12,13^

#### Real-Time Quantitative PCR (RT-qPCR) Analysis

To validate the differential gene expression of 13 selected genes (Table 1) obtained from Tag-seq analysis, qRTPCR was done using Power SYBR Green RNA-to-CT 1-step kit (Applied biosystems, USA). The primers used in this study were designed using the Primer-BLAST tool from NCBI. The data were normalized against the ACT1 gene. The double delta CT method was used to determine the fold change of genes between the control and treatment.

**Table 1:** List of Primers Used for qRTPCR Validation of Transcriptome Data Analysis

Primer name	Sequence 5′‘3′
CalPLB1	F:GGAGAGAGCAAGAAAGACAAG
	R:AATGGCTCACCCTTATAGATGG
Cal MSH6	F:TGCTGCAGGGAAATCGACAT
	R:TCCACAGGGGTCAATTCTGC
CalRNR1	F:ATCATGAGGGTTGCTGTCGG
	R:TGTGGTCTTGGTGTACCAGC
Cal PGA45	F:TGGGTAGATATTCTGGTGCCC
	R:TAGATGTAGCGGTGGCGGTA
CalYmL25	F:CACCACGACCATTTGCACAG
	R:AGAACTTTGCGCTTTGCCAG
CalPCK1	F:ATGTGATGCTTCCGGTGTGT
	R:TGACCGAAACATGCGGAGAA
CalAOX2	F:TGCTTACTGCTTCGCTTTACA
	R:TCCCACGGTATGAGCACAAT
CalCTR1	F:ATCAATGGTAACGTCGGCCA
	R:AACCGTGGTCCATACCTTCC
CalALS1	F:TACTTGTGCTGGCAGTCGTC
	R:ACCGTTAGATCCGGCATCAC
CalERG11	F:TCCAGTTTTCGGTAAAGGGGT
	R:ACATTGGCAACCCCATGAGT
CalERG5	F:CTGGCTCACCAATCACCACT
	R:AACGGGGACCAGCAATTGAA
CalACT1	F:TCTGTCGGCAGTGGTTTCAA
	R:GCCTTGCACAAGTACACGTAG

#### Filipin Analysis of Relative Cellular Ergosterol Levels

Filipin is a fluorescent probe that binds sterols and has been used to detect the relative levels of ergosterol in yeast. *C. albicans* exposed to control and NSS surfaces was examined at 2 h, which was the time for RNA collection for the TagSeq, and 18 h; all experiments were conducted at 25 °C. The rationale for these times is as follows: 2 h is the time of collection of the RNA and when permanent adhesions form, and 18 h represents a time that we observe a maximum effect of the NSS on the *Candida albicans*.^9^ Cells at each time point were labeled with a 1 μM Syto9 green nucleic acid stain (ex 485/em498; Thermofisher S34854) and 5 μg/mL filipin (340–380ex/285–407em; Sigma-Aldrich SAE0087). The cells were then fixed with 8% formaldehyde for 20 min, and the samples were imaged with a Zeiss Axio spinning disc confocal microscope using the 63× objective, the 405 nm laser line set at 80% power to excite the sample, and imaged under the 450-emission channel. The experiments were performed in triplicate. Intensity of the filipin fluorescence was measured using the ImageJ densitometric analysis toolset; statistical analysis was performed using Excel.

#### Antifungal Drug Assays

Two assays were used to determine changes to the Minimum Inhibitory Concentration (MIC) when *C. albicans* cells are exposed to the NSS. A CFU/dilution method in which *C. albicans* cells were cultured for 16 h on a glass surface or NSS in SBD medium that contained a range of drug concentrations: 0 (control), 0.5, 1, 1.5, 2.0, and 2.5 μg/mL. The following drugs were used in these experiments: Nystatin (ThermoFisher J67369.XF), Amphotericin B (Thermofisher J67049.AD), Fluconazole (Thermofisher 455480250). After 16 h of culture, the number of viable *C. albicans* cells were determined using a standard CFU assay. All the experiments were performed in triplicate and evaluated separately; significant reduction (*p* < 0.05) versus the untreated was compared to the no-drug treatment control. Changes to the MIC were also determined using commercially available MIC test strips: Liofilchem MTS Amphotericin B [AMB] 0.002–32 μg/mL (Fisher 22-777-981), Liofilchem MTS Fluconazole [FLU] 0.016–256 μg/mL (Fisher 22-777-965), Liofilchem MTS Micafungin [MYC] 0.002–32 μg/mL (Fisher 22-778-052). For these experiments, *C. albicans* cells were cultured either on control flat substrates or NSS for 16 h, after which the cells were plated onto an SBD agar medium with a test strip; the MIC was calculated as determined by the manufacturer.

## Results and Discussion

### Transcriptome Analysis C. albicans during Interaction with NSS

**Figure 1:**
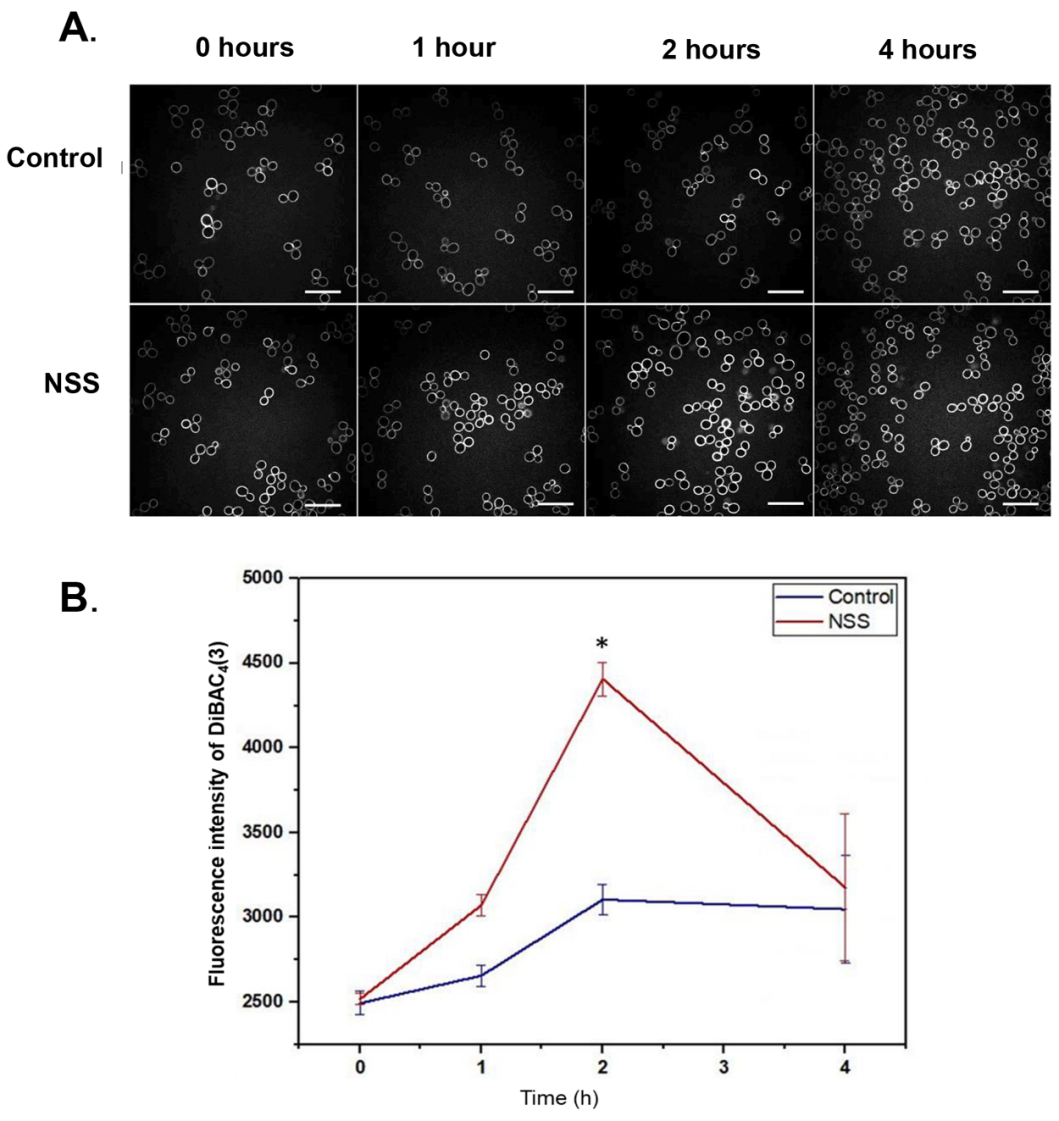
Alteration of plasma membrane potential in *C. albicans* cells cultured on NSS. (A) Confocal micrographs of *C. albicans* cells cultured on a flat control surface (top row) and on an NSS (bottom row) and labeled with DiBAC(4)3 dye. Images were collected at four time points, 0 h (prior to culture), 1, 2, and 4 h. Comparing the top to the bottom images, increased intensity of fluorescent sign is apparent at 2 h time point. (B) Graph showing densitometry of DiBAC(4)3 staining; collected from 30 cells per experiment over three independent experiments. NSS cells show a gradual increase in signal with the maximum difference at the 2 h mark, which is statically significant with a *P* value <0.001.

We performed a 3′ Tag-Sequencing (Tag-Seq) analysis of the *C. albicans* transcriptome to cells that has been exposed for 2 h to an NSS from the Tibiciens spp.’s cicada wing to define the differential NSS transcriptional response.^8,9^ Previous work demonstrated that the initial phenotypic response including the formation of permanent adhesion to the NSS began at 2 h.^9^ Furthermore, *C. albicans* cells when exposed to an NSS exhibited a dramatic change in membrane potential, as shown by DiBac(4)3 florescence. Wild-type *C. albicans* cells exposed to flat control surfaces exhibit little change in DiBac(4)3 membrane fluorescence (Figure 1A top row, 1B); however, *C. albicans* cells exposed to an NSS exhibit an increase in DiBac(4)3 fluorescence up 2 h after exposure (Figure 1A bottom row, 1B). In both control and experimental conditions, we observed no changes to chitin levels, as demonstrated by CFW staining (Supporting Information Figure 1). This result demonstrates that *C. albicans* contact with the NSS alters plasma membrane structure early and progressively during the first two hours of contact.

### TagSeq of 2 h *C. albicans* Transcriptome Response

We used the 2 h time point to examine changes to the *C. albicans* transcriptome when in contact with the NSS. To determine whether specific nanoscale material properties of the NSS contribute to the *C. albicans* NSS response, we examined gene expression changes in two separate control conditions. Cell-to-surface adhesion is a major component in biofilm formation and controls a wide range of cellular processes including metabolic and morphological switches in *C. albicans*.^14,15^ NSS, both native and synthetic, are self-cleaning/antiadhesive.^16−18^ To determine whether the antiadhesion nature of NSS contributes to a cellular NSS response, we examined the transcriptome of *C. albicans* cells exposed to reduced adhesive PEG-coated surfaces.^19,20^ Cellular interactions with NSS generate localized shear forces that are responsible for the cellular rupture in Gram-negative bacteria.^21,22^ To determine whether shear stresses also contribute to the phenotypes in *C. albicans* cells interacting with the NSS, we defined the transcriptome of *C. albicans* that had been exposed to a 10 mPa shearing event as generated by passing through a 1 mm silicon tube.

**Figure 2:**
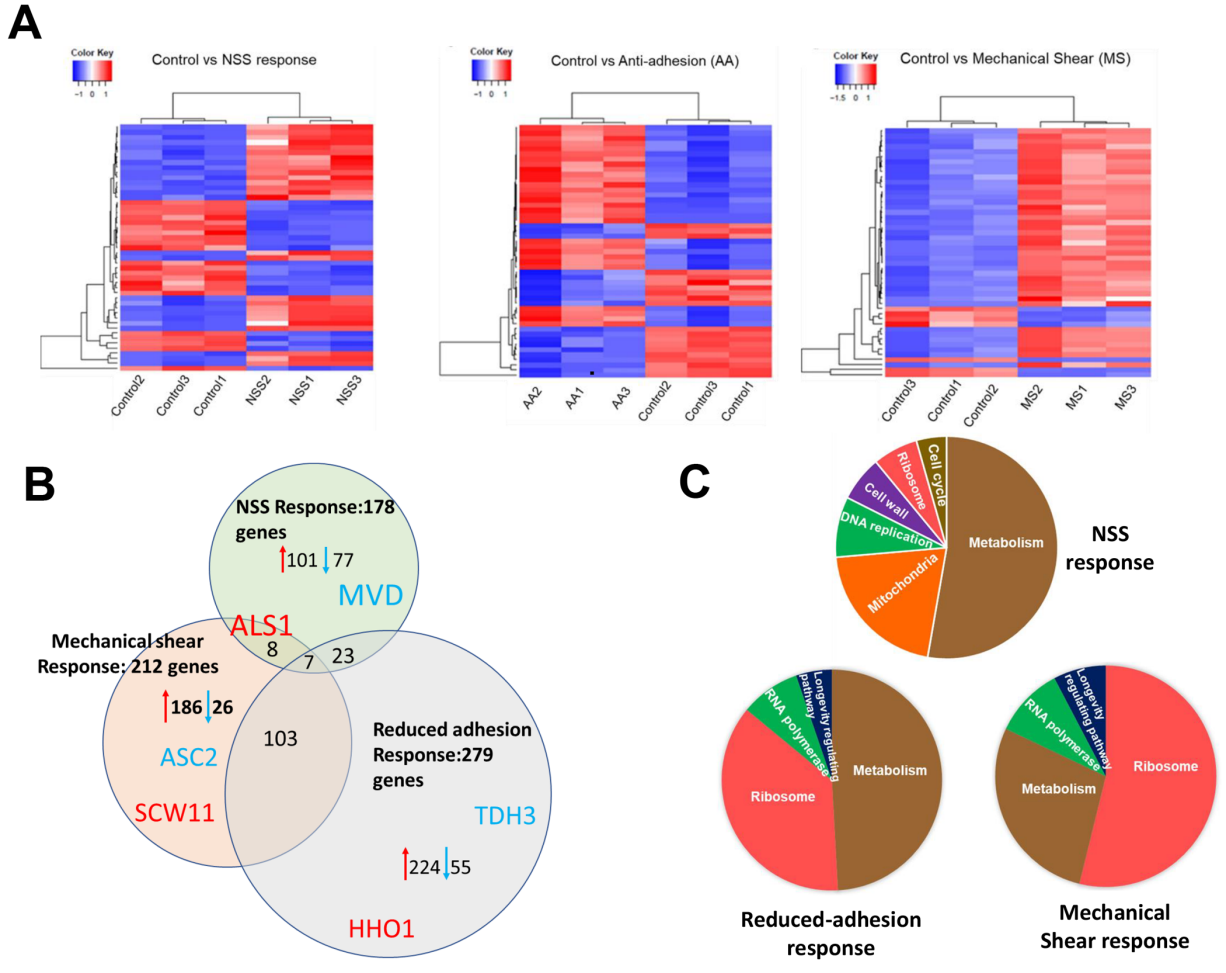
Summary of the TagSeq data. (A) Heat Maps of the top 50 genes for each condition for all three replicates when compared to the control. Left, NSS compared to control; Middle, Reduced adhesion; Right, mechanical shear. The colors signify the foldchange in expression; shades of red reflect increased expression compared to the control; blue indicates decreased expression. The consistency of each trial is reflected by clearly distinct blocks of shared values. (B) Pie graphs showing the overall distribution of gene categories for all three conditions that were identified in the TagSeq analysis. (C) Venn diagram of the relationship among all three conditions. In red we have listed the gene with the highest fold increase for each condition, and in blue we have listed the gene with the greatest fold decrease.

To determine the statistically significant genes in each treatment, we used a *P*-value cutoff of *p* < 0.01 and log2FC > 0.8 to focus on the genes with larger statistical change and compared the number of differentially expressed genes in each treatment to a non-nanostructured control (Table 2). A cluster heatmap demonstrates the consistency of each condition’s transcriptional response within each biological replicate: NSS response (Figure 2, left), reduced-adhesion response (Figure 2, middle), and mechanical shear response (Figure 2, right).

**Table 2:** 2 h NSS Response Genes

NSS response - Upregulated genes	
**Biological Function**	**Gene**
Mitochondria biosynthesis and protein expression	TOM40, YmL24/YmL14, YmL31, YmL35, MRPS17, MRPL19, YmL32, RSM27, RML2, YmL9, MGE1, YmL22, YmL17/YmL30, MRPS35, CAALFM_CR01370CA, RSM23,
	HSP60, SSC1, YmL25, YmL11, RSM7, MRPL10, MRPS9, TUF1, TIM23, TIM9, MRPL4, YmL23, YMR31, MAM33
Adhesion and biofilm formation	ALS1, COI1, HPT1
Cell cycle	SDS24
DNA repair	RAD16
Electron transport/ATP synthesis	ATP1, ATP4, ATP5, ATP20, QCR2, AOX2, RIP1, CYC1
Metabolism	SPE3, MAE1, FCA1, GUA1, CRG1, FUM12, YNK1, URA2, SOU1, PCK1
TCA cycle	KGD1, KGD2, SDH12, SDH2, IDH1, IDH2, ACO1, MDH1
Nitrosative/Oxidative Stress	YHB1, TRR1, GPX2, TSA1
Nuclear Import	MDN1, karyopherin beta
Transport	YHM1, PET9, MIR1, PAM18, FET34, CTR1, orf19.7077, FRE7, FTR2
Unknown	orf19.760, orf19.7215.3, orf19.5517, orf19.2275, orf19.93, orf19.6492, orf19.6090, orf19.670.2, orf19.2165, orf19.698, orf19.5114.1, orf19.1862, orf19.4450.1, orf19.35, orf19.2048, orf19.915, orf19.2414, orf19.7621, orf19.6853
**NSS response - Downregulated genes**	
**Biological Function**	**Gene**
Cell cycle, morphogenesis	SWE1, SMC1, CDC28, SUN41, SET3, PHO85
Metabolism	ACB1, FAS1, GDH2, GCV2, INO1, ACC1, FAS2, FAD2, DPP1, MTS1, PLB4.5, PLB1, SAM2, MET14, ECM17, MET3, SNZ1, MET15, TMP1, TDH3, PMI1, PGI1
Ergosterol Biosynthesis	ERG10, ERG5, ERG28, ERG11, MVD
DNA replication and repair,	POL3, TOP2, RFC4, RFA2, CAALFM_C505350WA, DDR48, GIN1, POL1, DUT1, POL2, RFA1, POL30, RNR1, MSH6
Stress response	YVC1, TCC1, CRZ2, PHO84, HMX1
RNA processing	SLT11, PAN2
Transport	ATO10, ALP1
Unknown	orf19.5510, orf19.4658, orf19.1691, orf19.3053, orf19.2123, orf19.7350, orf19.1964, orf19.4658, orf19.2452, orf19.5518, orf19.3793, orf19.33

Each condition expressed a distinct response to the NSS challenge with overlap between conditions, and seven genes were shared among all three (Figure 2B). When compared with the non-nanostructured surface control, we found 178 differentially expressed genes in the NSS response. Within these 178 genes of the NSS response, 101 genes were upregulated and 77 genes downregulated (Figure 2B, Table 3; Supporting Information Table 1). The mechanical shear response showed 212 differentially expressed genes with 185 upregulated and 27 downregulated; the antiadhesion response had 279 differential genes with 224 genes up-regulated and 55 genes down-regulated (Figure 2B, Supporting Information Table 1).

**Table 3:** Pathway Analysis of Differentially Expressed Genes in NSS, Reduced Adhesion, and Mechanical Shear Responses

Treatment	KEGG entry	Pathway	Count	% Frequency	EASE score
**NSS**	cal01200	Carbon metabolism	21	12.1	5.62 × 10^–05^
	cal00020	Citrate cycle (TCA cycle)	12	6.9	2.36 × 10^–05^
	cal00920	Sulfur metabolism	8	4.6	1.55 × 10^–04^
	cal03430	Mismatch repair	9	5.2	3.91 × 10^–04^
	cal03030	DNA replication	11	6.3	5.19 × 10^–04^
	cal00240	Pyrimidine metabolism	14	8.09	1.5 × 10^–03^
	cal00480	Glutathione metabolism	6	3.4	0.049
	cal00230	Purine metabolism	14	8.09	0.018
	cal03420	Nucleotide excision repair	9	5.2	0.011
	cal00190	Oxidative phosphorylation	13	7.5	0.0094
	cal01130	Biosynthesis of antibiotics	26	15.02	0.0043
	cal01110	Biosynthesis of secondary metabolites	27	15.6	0.078
**Reduced- adhesion response**	cal03008	Ribosome biogenesis	26	12.38	1.10 × 10^–13^
	cal03020	RNA polymerase	10	4.7	2.10 × 10^–04^
	cal00230	Purine metabolism	16	7.6	4.35 × 10^–04^
	cal00240	Pyrimidine metabolism	12	5.7	0.0042
	cal01200	Carbon metabolism	13	6.1	0.033
	cal00910	Nitrogen metabolism	4	1.9	0.0335
	cal03010	Ribosome	11	5.2	0.03
	cal00010	Glycolysis	4	1.9	0.0505
**Mechanical shear response**	cal03008	Ribosome biogenesis	22	14.1	2.12 × 10^–14^
	cal03020	RNA polymerase	7	4.4	0.0034
	cal00230	Purine metabolism	9	5.7	0.062

Among these three sets of experimental conditions, we hypothesized that any shared differentially regulated genes will signify a common response of *Candida albicans* to specific properties exhibited by these conditions. However, there was some overlap between the NSS conditions and the two controls, with the greatest shared genetic response between the cells’ mechanically stressed and reduced adhesive surfaces.

Between the NSS and Mechanical shear responses, we find nine genes common between the two conditions, six upregulated and three downregulated (Supporting Information Table 1). One of these genes is ALS1, which encodes an adhesion protein that belongs to large family of adhesion proteins^23−25^ and is a downstream target of one of the master biofilm regulators.^14,15^ The ALS1 was the highest upregulated transcription in the NSS response with an ∼16-fold increase and upregulated (∼2.8×) in the Mechanical shear response. Increased expression of the ALS1 gene supports previous work that demonstrates that cell–substrate adhesion is central to the NSS-induced rupture and suggests that mechanical stress is associated with ALS1 expression.^8^ The increase in ALS1 gene expression supports the notion that enhanced adhesion of *C. albicans* and other yeasts to the host surface may make the cell vulnerable to the surface stress and the enhanced expression of ALS1 may reflect the adaptation of the yeast cell during transition from a planktonic to sessile state. However, whether a common element of the two conditions, i.e., mechanical stress, is responsible for this upregulation between these conditions remains to be tested. Between the NSS and PEG-coated, reduced-adhesive conditions we found 23 overlapping genes. Here we observed the upregulation of seven genes associated with cell wall architecture/plasma membrane synthesis, which suggests that the NSS and the reduced adhesive surfaces may trigger a change in external cell structure, but more work will be needed to determine how these effects are manifested and whether they are triggered by a common response from the cell.

We found that the differentially expressed genes in the Mechanically Stressed cells and the reduced adhesion cells shared many genes with 103 genes in common (89 shared upregulated genes/14 downregulated genes), many of which reflect the shared increases in genes associated with catabolic metabolic pathways, including genes associated with protein production and ribosomal biosynthesis.

Seven differentially expressed genes were shared among all three conditions. Three genes were upregulated, and of these two, MRPS17 and Yml22, have homology to characterized genes, and one orf19.6060 encodes a novel/uncharacterized product. Both MRPS17 and Yml22 encode components of the mitochondrial ribosome and are involved in the mitochondrial protein production. Again, the significance of this upregulation is unclear. While all three conditions exhibited extensive alteration to genes associated with metabolic pathways, the Antiadhesion and the Mechanical Shear control conditions resulted in changes of genes expression of genes associated with anabolic pathways, including many genes that encoded components of the ribosome (Figure 2C, Supporting Information Table 2), while NSS response genes include a cluster of mitochondria biosynthetic genes and the downregulation of genes encoding metabolic pathway components (Figure 2C, Tables 2 & 3)

Four genes, SNZ1, PLB1, CRZ2, and a hypothetical product of unknown function ORF19.33 were downregulated (Table 2; Supporting Information Table 2). Three of these genes (SNZ1, CRZ2, and PLB1) are associated with biofilm formation, hyphal formation, and/or virulence.

SNZ1 encodes a component of pyridoxine biosynthesis, which is essential for Vitamin B6 production, which acts as an antioxidant and plays important roles in regulating the cell cycle, biofilm formation, and cell growth.^26,27^ PLB1 encodes Lysophospholipase 1 an enzyme that catalyzes the release of fatty acids from phospholipids and is associated with virulence.^28,29^ NSS surfaces have been demonstrated to inhibit hyphal formation, which is also associated with the virulent state of *C. albicans*(9) and suggests that preventing biofilm formation either through shear flow or reduced adhesion to a surface may contribute to lower levels of PLB1 expression. CRZ2 encodes a transcription factor that regulates pH-induced filamentation and is required for biofilm formation.^30,31^ While the overall NSS response of *C. albicans* shares little with Mechanical Shear and Reduced Adhesion responses, the response to the NSS by *C. albicans* cells is distinct. The physical interaction of yeast cells with NSS may share some similar responses, namely, reduced adhesion and/or mechanical shear.

### Gene Enrichment Analysis

Functional enrichment analysis was done on differentially expressed genes in NSS response using DAVID bioinformatics resources 6.8.^11,32^ The NSS response contained 12 classes of KEGG/GO process categories (Table 3). All of the differentially expressed genes in the NSS response, regardless of whether they were up- or down-regulated, fell into three Gene Ontology categories with EASE scores less than 0.1, and these categories are biological process (32 up/30 down-regulated), cellular component (16 up/7 downregulated), and molecular function (35 up/31 down-regulated). TAqSeq was validated by qRT-PCR analysis results of selected genes in which was shown a strong correlation (Pearson’s correlation *R* = 0.7612 with *p* ≤ 0.0043; Supporting Information Figure 2).

**Figure 3:**
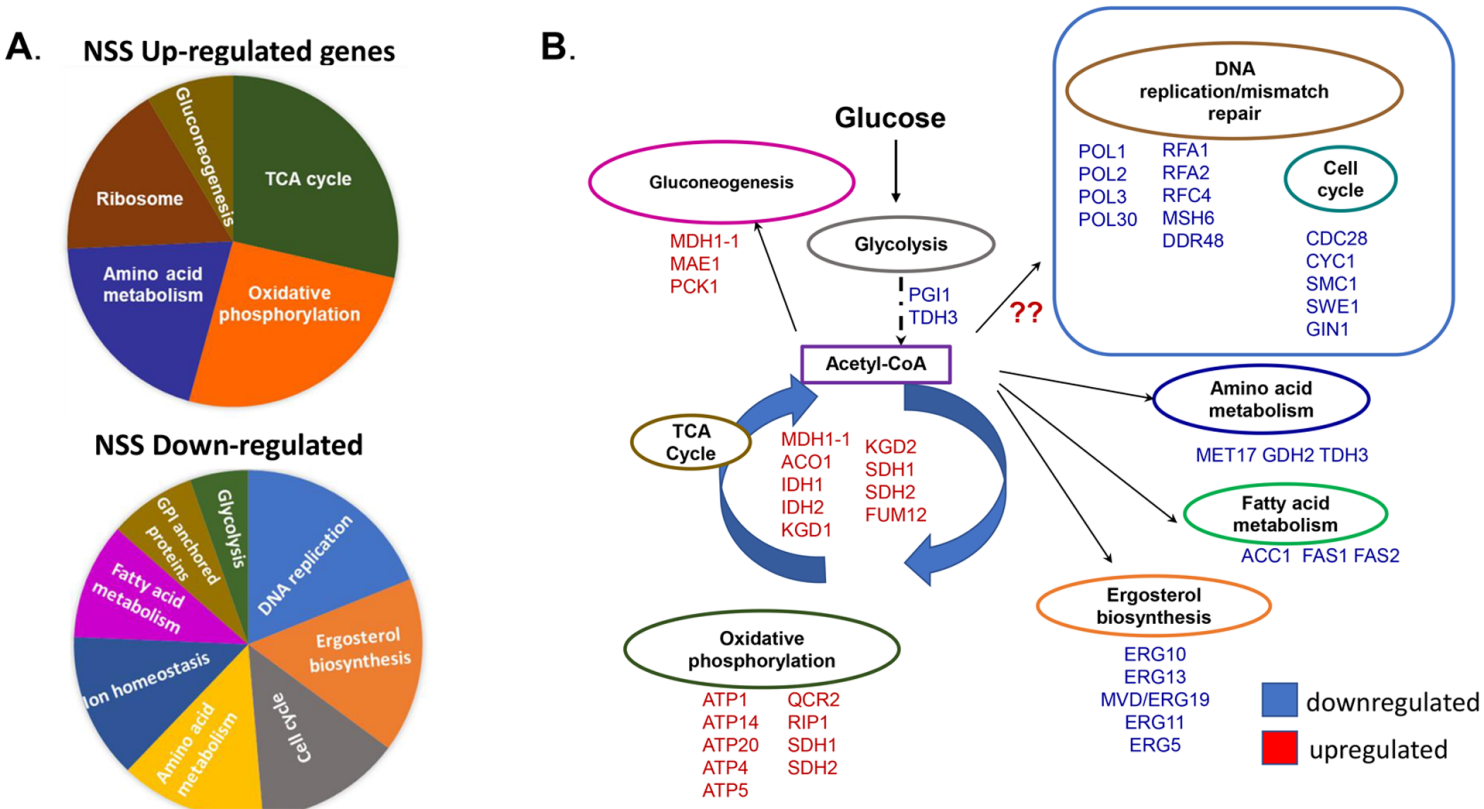
Summary of the NSS response. (A) Pie graphs showing relative proportion of gene classes that were upregulated (top) and downregulated (bottom). (B) Schematic of metabolism pathways potentially impacted by interactions with the NSS, red lettered gene names, upregulated, and blue lettered gene names, downregulated.

Previous work has shown that mechanical challenges to yeast cells result in the activation of specific signature transcriptional targets of signal transduction pathways such as the cell wall integrity pathway;^33,34^ however, we did not observe the expression of genes assocaited with any canonical stress-activated signaling pathway in the NSS response. The NSS response transcriptome enzyme contained genes with several metabolic pathways: ten metabolic pathways have genes that were up-regulated including the genes associated with the tricarboxylic acid (TCA) cycle, whereas genes associated with 14 metabolic pathways including the ergosterol biosynthesis pathway and DNA mismatch repair were down-regulated (Figure 3A; Tables 2, 3; Supporting Information Table 1). The pattern of gene expression in the NSS response suggests a metabolic shift to diauxic conditions (Figure 3B). Under stress conditions, *C. albicans* shifts the expression of genes associated with glycoslysis metabolic processes to genes associated with alternative carbon sources.^35,36^ These changes enable *C. albicans* to adapt to stress while increasing the biofilm formation and cell wall remodeling. Given the change we observe in the NSS trancriptome, we suggest that *C. albicans* cells may engage in a metabolic shift, as shown with the downregulation of glycolysis and upregulation in genes encoding compoenent of the tricarboxylic acid (TCA) cycle and mitochondrial biosynthesis (Figure 3B).^37^ Metabolic shifts have been observed in other systems including cancer and baker’s yeast;^38,39^ accordingly, we obsevered an ∼2 fold downregulation of the ACC1 gene which encodes the enzyme that catalyzes the acetyl-CoA carboxylation as have those other systems (Supporting Information Table 1); however, further assessment of the metabolic state of *C. albicans* cells cultured on NSS will need to be performed to confirm this hypothesis, but it does provide a potentially attractive possibilty to exploit for controlling biofilm formation. We also observe significant downregulation of genes associated with maintaining genomic stability in *C. albicans* cells in contact with an NSS (Table 2, Supporting Information Table 1). The role of this downregualtion is unclear; however, in other systems the loss of genomic stability has been shown to increase genetic diversity and potentially may be an adaptive mechanism to this stress.^40,41^ The observation suggests that NSS may also generate conditions in the cell that favorably lead to greater genetic diversity, which in turn may result in rapid evolution of adaptive traits such as drug resistance.

### NSS Response and the Ergosterol Biosynthetic Pathway Enzymes

**Figure 4:**
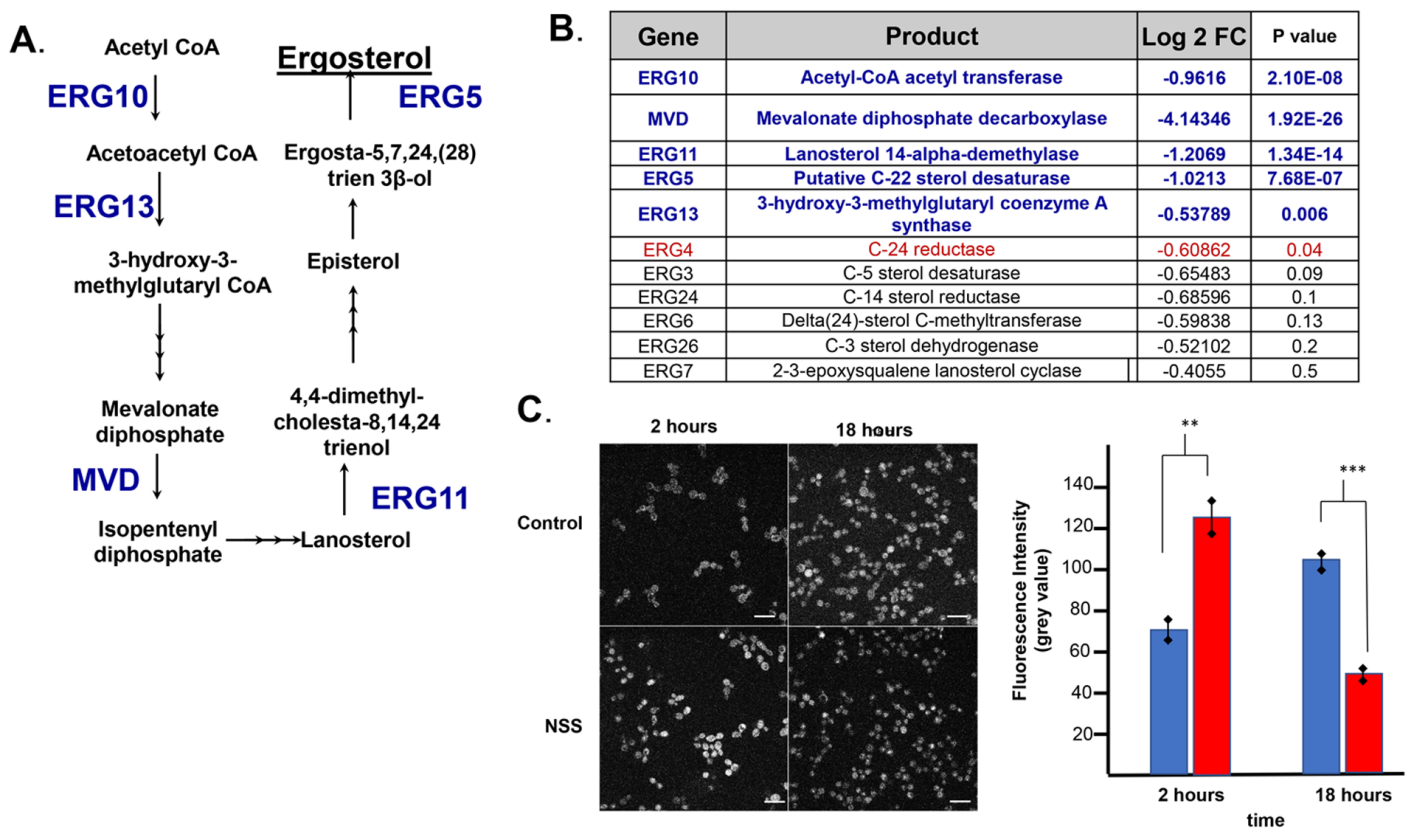
Impact of NSS on ergosterol biosynthesis. (A) Schematic of the ergosterol pathway; in blue letters are the genes encoding ergosterol biosynthesis and the reaction step in the pathway in which their product is catalyzed. (B) Table of all genes involved in the ergosterol biosynthesis pathway, their products, the log fold difference, and the *P* score. (C) Assessment of relative levels of cellular ergosterol using filipin staining. Left, representative confocal micrographs of *C. albicans* cells; controls top; NSS exposed bottom. Two time points 2 h - first column, and 18 h - second column. Right, a graph showing the densitometry analysis of ergosterol fluorescence in individual cells. ** *p* ≤ 0.002, *** *p* ≤ 0.00051.

Ergosterol is an essential component of the fungal plasma membrane that maintains plasma membrane integrity and certain signal transduction pathways.^39−42^ Ergosterol biosynthesis is a multistep pathway, and we observed the significant down-regulation five genes that encode enzymes within the ergosterol biosynthesis (Figure 4A) when *C. albcians* is cultured on an NSS. In addition to these five genes, we examined our transcriptome data and found six other genes that encode components of the ergosterol biosynthesis whose expression fell below either the signifcance or the expression thresholds (Figure 4B). To determine whether cellular levels of ergosterol were influenced by the NSS, we examined ergosterol in situ using the sterol labeling dye, flilipin.^42^ We observed that exposure to the NSS resulted in changes in cellular ergosterol levels in *C. albicans* (Figure 4C). At 2 h of exposure, levels of ergosterols increased in NSS exposed *C. albicans* cells relative to controls; however, after 18 h of exposure to an NSS we observe a signifcant decrease in cellular ergosterol relative to controls (Figure 4C). High levels of ergosterol results in decreased ergosterol biosynthesis due to the transcriptional downregualtion of ergosterol biosynthesis, which is controlled by a negative feedback loop that involves the sterol regulatory element binding proteins Upc2p, which negatively regulates the transcription of genes such as Erg11.^43,44^

**Figure 5:**
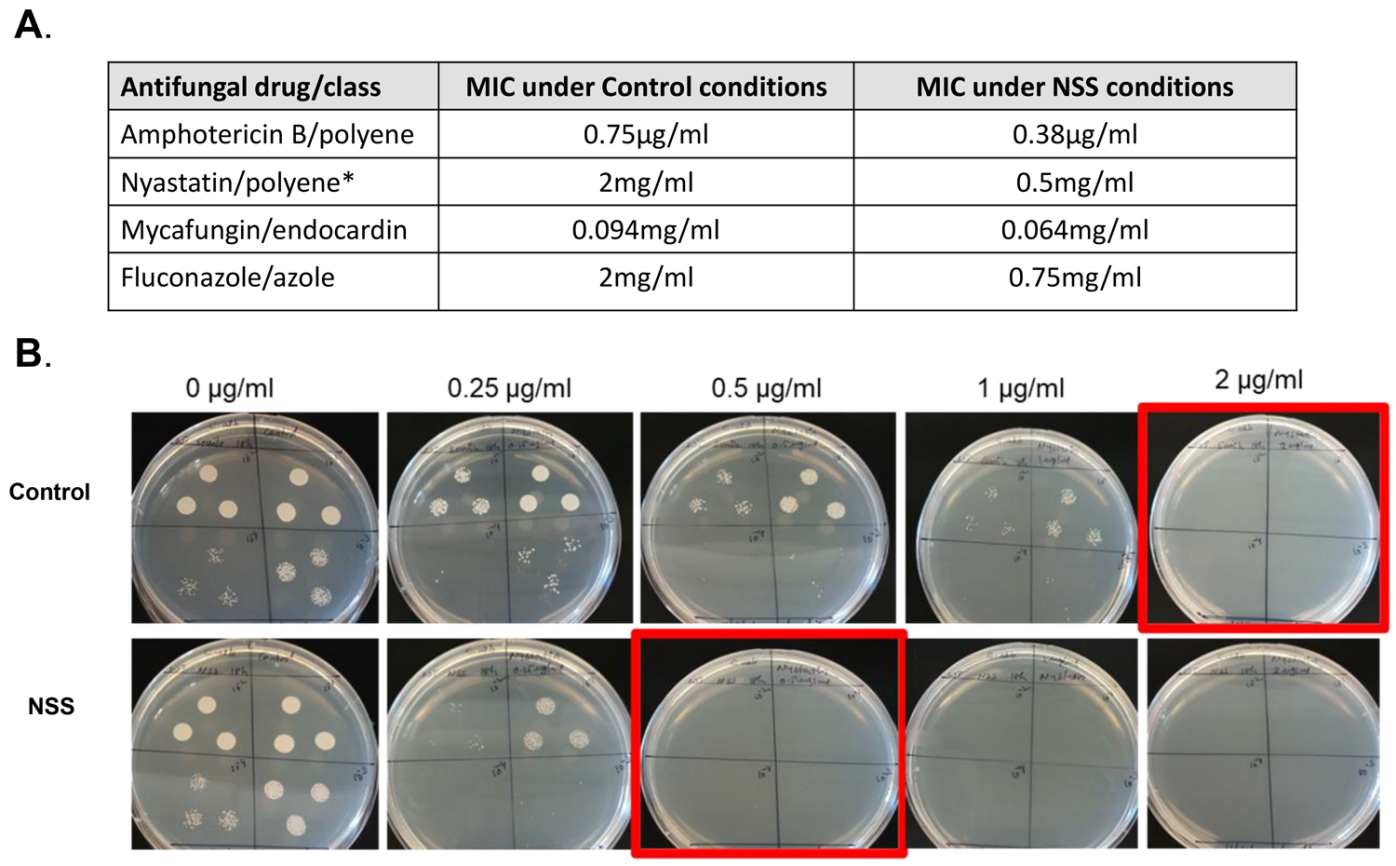
NSS exposure lowers the MIC for antifungal drugs. (A) Table showing MIC in control and NSS conditions. (*) Nystatin MIC was collected by CFU. (B) Example of CFU plates demonstrating Nystatin MIC reduction.

Several classes of antifungal drugs target ergosterol production (i.e., azoles) or directly sequester ergosterol from the cell membrane (i.e., polyenes).^45^ To determine of whether the alteration in ergosterol biosynthesis alters the response of *C. albicans* to antifungal drugs we measured the MIC of several antifungal drugs in cells exposed to an NSS when compared to control (Figure 5). We found that culturing of *C. albicans* on the NSS resulted in the cells that had a lower MIC for the polyenes, amphotericin B and nystatin, and the azole, fluconazole.

Interestingly we also found that the NSS also sensitized the *C. albicans* to the echinochandin, micafungin. These results suggest that the impact on the *C. albicans* cell may include a challenge to both the plasma membrane and later the cell wall as well. NSS has been shown to alter levels of chitin in the cell wall and echinocandins target the biosynthesis of cell wall glucans; perhaps the action of the NSS weakens the cell wall/plasma membrane and in some cases results in rupture.

## Conclusion

Using TagSeq, we defined the transcriptome of *C. albicans* cells when cultured on an NSS and identified 178 genes. When compared to a control that deconstructs the material properties of an NSS, specific reduced adhesion and mechanical shear stress, we find some overlap but significant difference. The transcriptome of the NSS cultured cells is marked by the downregulation of genes encoding essential metabolic processes that suggest that these yeast cells are engaging a metabolic or diauxic shift. Furthermore, we find that the NSS response transcriptome also has reduced expression of genes associated with DNA repair suggesting that perhaps these cells are adapting to this stress by increasing the genetic diversity. Of the downregulated metabolic processes, we found that five genes encoding the ergosterol biosynthesis pathways including Erg11, the target of the azole class of antifungal drugs, are downregulated. We show that this results in a reduction of cellular ergosterol and the sensitization of these cells to several classes of antifungal drugs including those that target ergosterol, such as the azoles and polyenes. These results suggest that nanoscale mechanical perturbations may be used to control fungal biofilm and extend the use of current antifungal drugs.

## Data Availability

The sequencing files containing all the raw data have been submitted to NCBI and can be found via the BioProject, PRJNA1023583, http://www.ncbi.nlm.nih.gov/bioproject/1023583.

## Supporting Information Available

Figure 1: CFW labeling of *C. albicans* exposed to control and NSS surfaces and Figure 2: Validation of Tag-Seq analysis using qRT-PCR. Tables 1–6 contain lists of all the differentially expressed genes. Table 7 contains a list of genes shared among two or all the groups (PDF)

## References

[ref1] Iyer K. R.; Robbins N.; Cowen L.E. The role of Candida albicans stress response pathways in antifungal tolerance and resistance. iScience 2022, 25 (3), 103953 10.1016/j.isci.2022.103953. 35281744 PMC8905312

[ref2] Lee Y.; et al. Antifungal Drug Resistance: Molecular Mechanisms in Candida albicans and beyond. Chem. Rev. 2021, 121 (6), 3390–3411. 10.1021/acs.chemrev.0c00199. 32441527 PMC8519031

[ref3] Aburto-Medina A.; Le P. H.; MacLaughlin S.; Ivanova E. Diversity of experimental designs for the fabrication of antifungal surfaces for the built environment. Appl. Microbiol. Biotechnol. 2021, 105 (7), 2663–2674. 10.1007/s00253-021-11214-0. 33704514

[ref4] Ivanova E. P.; Linklater D. P.; Aburto-Medina A.; Le P.; Baulin V. A.; Khuong Duy Nguyen H.; Curtain R.; Hanssen E.; Gervinskas G.; Hock Ng S.; Khanh Truong V.; Luque P.; Ramm G.; Wösten H. A. B.; Crawford R. J.; Juodkazis S.; Maclaughlin S. Antifungal versus antibacterial defence of insect wings. J. Colloid Interface Sci. 2021, 603, 886–897. 10.1016/j.jcis.2021.06.093. 34265480

[ref5] Linklater D. P.; Le P. H.; Aburto-Medina A.; Crawford R. J.; Maclaughlin S.; Juodkazis S.; Ivanova E. P. Biomimetic Nanopillar Silicon Surfaces Rupture Fungal Spores. International Journal of Molecular Sciences 2023, 24 (2), 1298 10.3390/ijms24021298. 36674814 PMC9864238

[ref6] Ivanova E. P.; Hasan J.; Webb H. K.; Truong V. K.; Watson G. S.; Watson J. A.; Baulin V. A.; Pogodin S.; Wang J. Y.; Tobin M. J.; Löbbe C.; Crawford R. J. Natural bactericidal surfaces: Mechanical rupture of pseudomonas aeruginosa cells by cicada wings. Small 2012, 8 (16), 2489–2494. 10.1002/smll.201200528. 22674670

[ref7] Linklater D. P.; Ivanova E.P. Nanostructured antibacterial surfaces – What can be achieved?. Nano Today 2022, 43, 101404 10.1016/j.nantod.2022.101404.

[ref8] Nowlin K.; Boseman A.; Covell A.; LaJeunesse D. Adhesion-dependent rupturing of Saccharomyces cerevisiae on biological antimicrobial nanostructured surfaces. J. R Soc. Interface 2015, 12 (102), 20140999 10.1098/rsif.2014.0999. 25551144 PMC4277089

[ref9] Kollu N. V.; LaJeunesse D. R. Cell Rupture and Morphogenesis Control of the Dimorphic Yeast Candida albicans by Nanostructured Surfaces. ACS Omega 2021, 6 (2), 1361–1369. 10.1021/acsomega.0c04980. 33490795 PMC7818643

[ref10] Afgan E.; Baker D.; Batut B.; Van Den Beek M.; Bouvier D.; Cech M.; Chilton J.; Clements D.; Coraor N.; Grüning B. A.; Guerler A.; Hillman-Jackson J.; Hiltemann S.; Jalili V.; Rasche H.; Soranzo N.; Goecks J.; Taylor J.; Nekrutenko A.; Blankenberg D. The Galaxy platform for accessible, reproducible and collaborative biomedical analyses: 2018 update. Nucleic Acids Res. 2018, 46 (W1), W537–W544. 10.1093/nar/gky379. 29790989 PMC6030816

[ref11] Huang D. W.; Sherman B. T.; Lempicki R. A. Bioinformatics enrichment tools: Paths toward the comprehensive functional analysis of large gene lists. Nucleic Acids Res. 2009, 37 (1), 1–13. 10.1093/nar/gkn923. 19033363 PMC2615629

[ref12] Kanehisa M.; Goto S. KEGG: Kyoto Encyclopedia of Genes and Genomes. Nucleic Acids Res. 2000, 28 (1), 27–30. 10.1093/nar/28.1.27. 10592173 PMC102409

[ref13] Kanehisa M.; Furumichi M.; Sato Y.; Ishiguro-Watanabe M.; Tanabe M. KEGG: Integrating viruses and cellular organisms. Nucleic Acids Res. 2021, 49 (D1), D545–D551. 10.1093/nar/gkaa970. 33125081 PMC7779016

[ref14] Nobile C. J.; Fox E. P.; Nett J. E.; Sorrells T. R.; Mitrovich Q. M.; Hernday A. D.; Tuch B. B.; Andes D. R.; Johnson A. D. A recently evolved transcriptional network controls biofilm development in Candida albicans. Cell 2012, 148 (1–2), 126–138. 10.1016/j.cell.2011.10.048. 22265407 PMC3266547

[ref15] Mancera E.; Nocedal I.; Hammel S.; Gulati M.; Mitchell K. F.; Andes D. R.; Nobile C. J.; Butler G.; Johnson A. D. Evolution of the complex transcription network controlling biofilm formation in candida species. eLife, 2021. 10. 10.7554/eLife.64682 PMC8075579 33825680

[ref16] Sun M.; Watson G. S.; Zheng Y.; Watson J. A.; Liang A. Wetting properties on nanostructured surfaces of cicada wings. Journal of Experimental Biology 2009, 212 (19), 3148–55. 10.1242/jeb.033373. 19749108

[ref17] Watson G. S.; Cribb B. W.; Watson J. A. How micro/nanoarchitecture facilitates anti- wetting: an elegant hierarchical design on the termite wing. ACS Nano 2010, 4 (1), 129–36. 10.1021/nn900869b. 20099910

[ref18] Watson G. S.; Cribb B. W.; Watson J. A. The role of micro/nano channel structuring in repelling water on cuticle arrays of the lacewing. J. Struct. Biol. 2010, 171 (1), 44–51. 10.1016/j.jsb.2010.03.008. 20347993

[ref19] Xu L. C.; Siedlecki C.A. Surface texturing and combinatorial approaches to improve biocompatibility of implanted biomaterials. Frontiers in Physics, 2022. 10. 10.3389/fphy.2022.994438 PMC10798815 38250242

[ref20] Caro A.; Humblot V.; Methivier C.; Minier M.; Salmain M.; Pradier C. M. Grafting of Lysozyme and/or Poly(ethylene glycol) to Prevent Biofilm Growth on Stainless Steel Surfaces. J. Phys. Chem. B 2009, 113 (7), 2101–2109. 10.1021/jp805284s. 19166331

[ref21] Ivanova E. P.; Linklater D. P.; Werner M.; Baulin V. A.; Xu X.; Vrancken N.; Rubanov S.; Hanssen E.; Wandiyanto J.; Truong V. K.; Elbourne A.; Maclaughlin S.; Juodkazis S.; Crawford R. J. The multi-faceted mechano-bactericidal mechanism of nanostructured surfaces. Proc. Natl. Acad. Sci. U.S.A. 2020, 117 (23), 12598–12605. 10.1073/pnas.1916680117. 32457154 PMC7293705

[ref22] Lohmann S. C.; Tripathy A.; Milionis A.; Keller A.; Poulikakos D. Effect of Flexibility and Size of Nanofabricated Topographies on the Mechanobactericidal Efficacy of Polymeric Surfaces. ACS Applied Bio Materials 2022, 5 (4), 1564–1575. 10.1021/acsabm.1c01318. 35176858

[ref23] Hoyer L. L.; Scherer S.; Shatzman A. R.; Livi G. P. Candida albicans ALS1: domains related to a Saccharomyces cerevisiae sexual agglutinin separated by a repeating motif. Mol. Microbiol. 1995, 15 (1), 39–54. 10.1111/j.1365-2958.1995.tb02219.x. 7752895

[ref24] Loza L.; Fu Y.; Ibrahim A. S.; Sheppard D. C.; Filler S. G.; Edwards J. E. Jr. Functional analysis of the Candida albicans ALS1 gene product. Yeast 2004, 21 (6), 473–82. 10.1002/yea.1111. 15116430

[ref25] Zhao X.; Oh S.-H.; Coleman D. A.; Hoyer L. L. ALS1 Deletion Increases the Proportion of Small Cells in a Candida albicans Culture Population: Hypothesizing a Novel Role for Als1. Frontiers in Cellular and Infection Microbiology, 2022. 12. 10.3389/fcimb.2022.895068 PMC9130707 35646731

[ref26] Uppuluri P.; Sarmah B.; Chaffin W. L. Candida albicans SNO1 and SNZ1 expressed in stationary-phase planktonic yeast cells and base of biofilm. Microbiology 2006, 152 (7), 2031–2038. 10.1099/mic.0.28745-0. 16804178

[ref27] Rodríguez-Navarro S.; Llorente B.; Rodríguez-Manzaneque M. T.; Ramne A.; Uber G.; Marchesan D.; Dujon B.; Herrero E.; Sunnerhagen P.; Pérez-Ortín J. E. Functional analysis of yeast gene families involved in metabolism of vitamins B1 and B6. Yeast 2002, 19 (14), 1261–1276. 10.1002/yea.916. 12271461

[ref28] Cho T. Virulence factors of the fungal pathogen Candida albicans. Japanese Journal of Medical Mycology 2009, 50 (3), 179–185. 10.3314/jjmm.50.179. 19654452

[ref29] Remmele C. W.; Luther C. H.; Balkenhol J.; Dandekar T.; Müller T.; Dittrich M. T. Integrated inference and evaluation of host-fungi interaction networks. Frontiers in Microbiology 2015, 6 ( (AUG), ). 10.3389/fmicb.2015.00764 PMC4523839 26300851

[ref30] Bensen E. S.; Martin S. J.; Li M.; Berman J.; Davis D. A. Transcriptional profiling in Candida albicans reveals new adaptive responses to extracellular pH and functions for Rim101p. Mol. Microbiol. 2004, 54 (5), 1335–1351. 10.1111/j.1365-2958.2004.04350.x. 15554973

[ref31] Karababa M.; Valentino E.; Pardini G.; Coste A. T.; Bille J.; Sanglard D. CRZ1, a target of the calcineurin pathway in Candida albicans. Mol. Microbiol. 2006, 59 (5), 1429–1451. 10.1111/j.1365-2958.2005.05037.x. 16468987

[ref32] Huang D. W.; Sherman B. T.; Lempicki R. A. Systematic and integrative analysis of large gene lists using DAVID bioinformatics resources. Nat. Protoc. 2009, 4 (1), 44–57. 10.1038/nprot.2008.211. 19131956

[ref33] Du J.; Dong Y.; Zhao H.; Peng L.; Wang Y.; Yu Q.; Li M. Transcriptional regulation of autophagy, cell wall stress response and pathogenicity by Pho23 in C. albicans. FEBS Journal 2023, 290 (3), 855–871. 10.1111/febs.16636. 36152022

[ref34] da Silva Dantas A.; Nogueira F.; Lee K. K.; Walker L. A.; Edmondson M.; Brand A. C.; Lenardon M. D.; Gow N. A. R. Crosstalk between the calcineurin and cell wall integrity pathways prevents chitin overexpression in Candida albicans. J. Cell Science 2021, 134 ( (24), ). 10.1242/jcs.258889 PMC8729787 34792152

[ref35] Vivier M. A.; Lambrechts M. G.; Pretorius I. S. Coregulation of starch degradation and dimorphism in the yeast Saccharomyces cerevisiae. Crit. Rev. Biochem. Mol. Biol. 1997, 32 (5), 405–435. 10.3109/10409239709082675. 9383611

[ref36] Uppuluri P.; Chaffin W. L. Defining Candida albicans stationary phase by cellular and DNA replication, gene expression and regulation. Mol. Microbiol. 2007, 64 (6), 1572–1586. 10.1111/j.1365-2958.2007.05760.x. 17555439

[ref37] Tao L.; Zhang Y.; Fan S.; Nobile C. J.; Guan G.; Huang G. Integration of the tricarboxylic acid (TCA) cycle with cAMP signaling and Sfl2 pathways in the regulation of CO2 sensing and hyphal development in Candida albicans. PLoS Genet 2017, 13 (8), e1006949 10.1371/journal.pgen.1006949. 28787458 PMC5567665

[ref38] Shiratori R.; Furuichi K.; Yamaguchi M.; Miyazaki N.; Aoki H.; Chibana H.; Ito K.; Aoki S. Glycolytic suppression dramatically changes the intracellular metabolic profile of multiple cancer cell lines in a mitochondrial metabolism-dependent manner. Sci. Rep 2019, 9 (1), 18699 10.1038/s41598-019-55296-3. 31822748 PMC6904735

[ref39] Al-Feel W.; DeMar J. C.; Wakil S. J. A Saccharomyces cerevisiae mutant strain defective in acetyl-CoA carboxylase arrests at the G2/M phase of the cell cycle. Proc. Natl. Acad. Sci. U. S. A. 2003, 100 (6), 3095–100. 10.1073/pnas.0538069100. 12626751 PMC152252

[ref40] Rizzo M.; Soisangwan N.; Vega-Estevez S.; Price R. J.; Uyl C.; Iracane E.; Shaw M.; Soetaert J.; Selmecki A.; Buscaino A. Stress combined with loss of the Candida albicans SUMO protease Ulp2 triggers selection of aneuploidy via a two-step process. PLoS Genetics 2022, 18 (12), e1010576 10.1371/journal.pgen.1010576. 36574460 PMC9829183

[ref41] Mba I. E.; Nweze E. I.; Eze E. A.; Anyaegbunam Z. K. G. Genome plasticity in Candida albicans: A cutting-edge strategy for evolution, adaptation, and survival. Infection, Genetics and Evolution 2022, 99, 105256 10.1016/j.meegid.2022.105256. 35231665

[ref42] Martin S. W.; Konopka J. B. Lipid raft polarization contributes to hyphal growth in Candida albicans. Eukaryotic Cell 2004, 3 (3), 675–684. 10.1128/EC.3.3.675-684.2004. 15189988 PMC420133

[ref43] Znaidi S.; Weber S.; Zin Al-Abdin O.; Bomme P.; Saidane S.; Drouin S.; Lemieux S.; De Deken X.; Robert F.; Raymond M. Genomewide location analysis of Candida albicans Upc2p, a regulator of sterol metabolism and azole drug resistance. Eukaryotic Cell 2008, 7 (5), 836–847. 10.1128/EC.00070-08. 18390649 PMC2394978

[ref44] Hoot S. J.; Oliver B. G.; White T. C. Candida albicans UPC2 istranscriptionally induced in response to antifungal drugs and anaerobicity through Upc2p-dependent and-independent mechanisms. Microbiology 2008, 154 (9), 2748–2756. 10.1099/mic.0.2008/017475-0. 18757808 PMC2577385

[ref45] Bhattacharya S.; Sae-Tia S.; Fries B. C. Candidiasis and mechanisms of antifungal resistance. Antibiotics 2020, 9 (6), 312 10.3390/antibiotics9060312. 32526921 PMC7345657

