# Supplementary material for: Transcriptional Response of Candida albicans to Nanostructured Surfaces Provides Insight into Cellular Rupture and Antifungal Drug Sensitization
Source: ACS Biomater Sci Eng. 2023 Nov 17;9(12):6724–33. doi: 10.1021/acsbiomaterials.3c00938 (PMC10716851; doi:10.1021/acsbiomaterials.3c00938)
Supplement: Supplementary file 1 — ab3c00938_si_001.pdf [file ab3c00938_si_001.pdf]

Supplemental Material

Transcriptional Response of Candida albicans to Nanostructured Surfaces Provides Insight into Cellular Rupture and Antifungal drug sensitization.

Lakshmi Gayitri Chivukula and Dennis LaJeunesse*

Department of Nanoscience, Joint School of Nanoscience and Nanoengineering, University of North Carolina Greensboro, 2907 East Lee Street, Greensboro, North Carolina, 27455

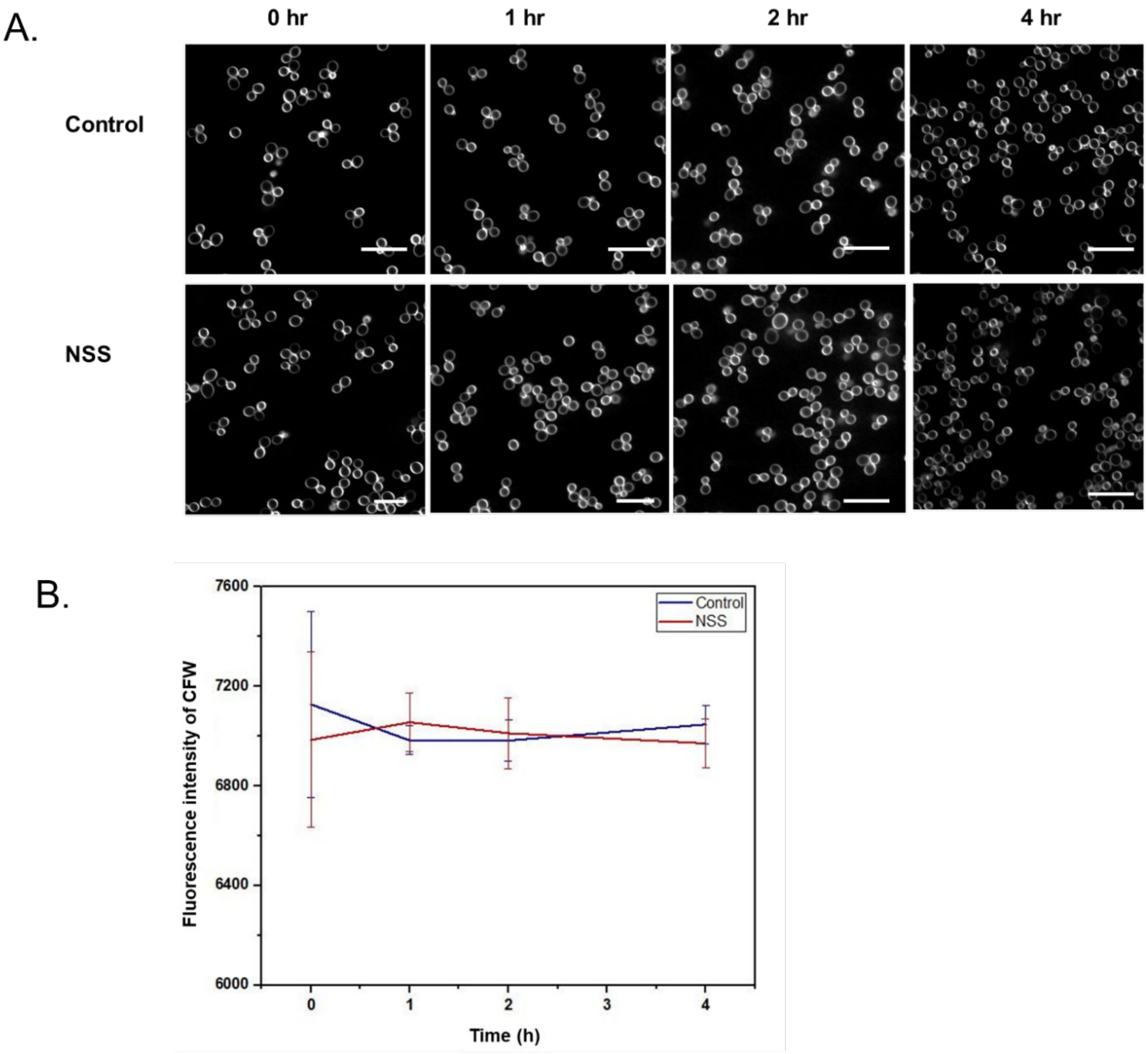

Supplemental Figure 1: CFW labeling of *C. albicans* exposed to control and NSS surfaces. A) Confocal micrographs of *C. albicans* cells culture on a flat control surface (top row) and on a NSS (bottom row) and labeled with CFW dye which labeled cell wall chitin. Images were collected at four time points, 0 hour (prior to culture), 1hr, 2hr and 4 hr. Comparing top to the

bottom images, there is no change in intensity of fluorescent signal. B) Graph showing densitometry of CFW staining. No difference between NSS and control.

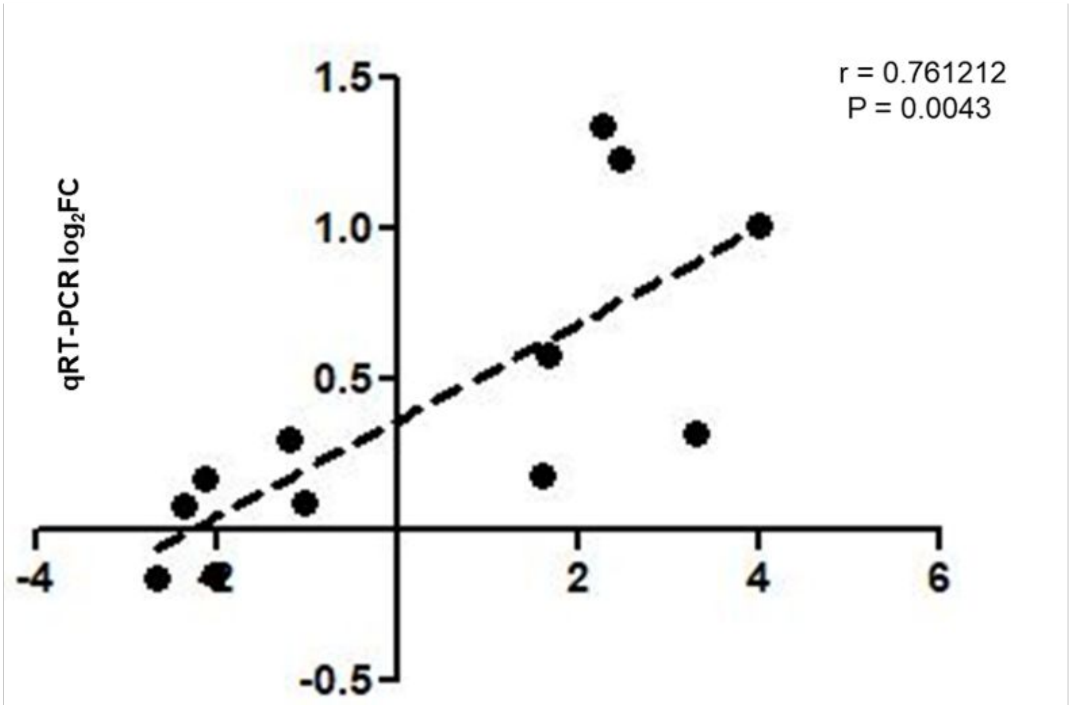

Supplemental Figure 2: validation of Tag-Seq analysis using qRT-PCR.

Supplemental Tables:

Supplemental Table 1: Downregulated genes in *C. albicans* NSS response

Gene Name	ORF	log FC	Product	Localization	Biological Process
MVD	orf19.6105	-4.143	Diphosphomevalonate decarboxylase	endoplasmic reticulum	metabolism - ergosterol biosynthesis
PLB1	orf19.689	-2.673	Lysophospholipase 1	extracellular, biofilm	metabolism - phospholipid catalysis
MSH6	orf19.4945	-2.376	DNA mismatch repair protein	nucleus	DNA replication and repair
PAN2	orf19.4764	-2.229	PAN2-PAN3 deadenylation complex catalytic subunit PAN2	cytoplasm	RNA processing
RNR1	orf19.5779	-2.145	Ribonucleoside-diphosphate reductase	nucleus	DNA replication and repair
PGA45	orf19.2451	-2.021	Predicted GPI-anchored protein 45	cell membrane, cell wall	cell wall/biofilm formation
POL30	orf19.4616	-1.933	Proliferating cell nuclear antigen	nucleus	DNA replication and repair
ECM331	orf19.4255	-1.900	Cell surface GPI-anchored protein ECM33	cell wall	cell wall/biofilm formation
SNZ1	orf19.2947	-1.819	Pyridoxal 5'-phosphate synthase	cytoplasm	metabolism - sulfur amino acid biosynthesis
RFA1	orf19.2093	-1.808	Replication protein A subunit	nucleus	DNA replication and repair
PGA10	orf19.5674	-1.774	GPI-anchored hemophore PGA10	plasma membrane	iron homeostasis
SMC1	orf19.4367	-1.771	Structural maintenance of chromosomes protein	nucleus	cell cycle, morphogenesis
MET3	orf19.5025	-1.742	Sulfate adenylyltransferase	cytosol	metabolism - sulfur amino acid biosynthesis
PLB4.5	orf19.1442	-1.736	Lysophospholipase	extracellular, biofilm, cell wall	metabolism - phospholipid catalysis
POL2	orf19.2365	-1.710	DNA polymerase epsilon catalytic subunit	nucleus	DNA replication and repair
MNN22	orf19.3803	-1.671	Alpha-1,2-mannosyltransferase MNN22	golgi apparatus	cell wall/biofilm formation
DUT1	orf19.3322	-1.670	Deoxyuridine 5'-triphosphate nucleotidohydrolase	nucleus	DNA replication and repair
POL1	orf19.5873	-1.648	DNA polymerase	nucleus	DNA replication and repair

Hypothetical	orf19.1691	-1.648	HIG1 domain-containing protein	plasma membrane, mitochondria*	unknown
Hypothetical	orf19.33	-1.632	Uncharacterized protein	unknown	unknown
MET15	orf19.5645	-1.621	Bifunctional cysteine synthase	extracellular	metabolism - sulfur amino acid biosynthesis
GIN1	orf19.658	-1.542	Chromatin-modulating protein	nucleus	DNA replication and repair
Hypothetical	orf19.3793	-1.495	Uncharacterized protein	unknown	unknown
FAS2	orf19.5949	-1.479	Fatty acid synthase subunit alpha	cytoplasm	metabolism - lipid/fatty acid biosynthesis
FAS1	orf19.979	-1.411	Fatty acid synthase subunit beta	cytoplasm	metabolism - fatty acid biosynthesis
HMX1	orf19.6073	-1.410	Hmx1p	endoplasmic reticulum, plasma membrane	iron homeostasis, stress response
DDR48	orf19.4082	-1.405	Stress protein DDR48	nucleus	DNA replication and repair
RFA2	orf19.2267	-1.373	Rfa2p	nucleus	DNA replication and repair
	orf19.4030	-1.368	DNA primase	nucleus	DNA replication and repair
Hypothetical	orf19.5518	-1.360	Uncharacterized protein	unknown	unknown
TMP1	orf19.3549	-1.304	Thymidylate synthase	cytoplasm	Metabolism - TTP/TMP biosynthesis
Hypothetical	orf19.2452	-1.254	Uncharacterized protein	unknown	unknown
RFC4	orf19.7658	-1.250	Replication factor C subunit 4	nucleus, cytoplasm	DNA replication and repair
ECM17	orf19.4099	-1.232	Sulfite reductase (NADPH) subunit beta	endoplasmic reticulum	metabolism - sulfur amino acid biosynthesis
FAD2	orf19.118	-1.212	Fad2p	membrane	metabolism - lipid/fatty acid biosynthesis
Hypothetical	orf19.4658	-1.212	Uncharacterized protein	nucleus*	unknown
ERG11	orf19.922	-1.207	Lanosterol 14-alpha demethylase	endoplasmic reticulum	metabolism - ergosterol biosynthesis
INO1	orf19.7585	-1.197	Inositol-3-phosphate synthase	cytoplasm	metabolism - lipid/fatty acid
TOP2	orf19.2873	-1.167	DNA topoisomerase 2	nucleus	DNA replication and repair
ERG28	orf19.2016	-1.159	Ergosterol biosynthetic protein 28	endoplasmic reticulum	metabolism - ergosterol biosynthesis
Hypothetical	orf19.1964	-1.159	Uncharacterized protein	membranes	unknown

GDH2	orf19.2192	-1.127	NAD-specific glutamate dehydrogenase	mitochondria	metabolism - glutamate catalysis
Hypothetical	orf19.4658	-1.106	Uncharacterized protein	nucleus*	unknown
POL3	orf19.5182	-1.104	DNA polymerase delta catalytic subunit	nucleus	DNA replication and repair
RCT1	orf19.7350	-1.082	Rct1p	plasma membrane, extracellular matrix	unknown
PGA54	orf19.2685	-1.081	Predicted GPI-anchored protein 54	cell wall/membrane	cell wall/biofilm formation
PMI1	orf19.1390	-1.062	Mannose-6-phosphate isomerase	cytoplasm	metabolism - carbohydrate catalysis
CRZ2	orf19.2356	-1.055	Transcriptional regulator CRZ2	nucleus	stress response - pH, copper, adhesion; morphogenesis; biofilm
GCV2	orf19.385	-1.053	Glycine cleavage system P protein	mitochondria	metabolism - glycine catalysis
MET14	orf19.946	-1.050	Adenylyl-sulfate kinase	cytoplasm	metabolism - sulfur amino acid biosynthesis
SET3	orf19.7221	-1.042	Histone-binding protein	nucleus	cell cycle, morphogenesis
Hypothetical	orf19.2123	-1.042	Uncharacterized protein	unknown	unknown
ALP1	orf19.2337	-1.028	Alp1p	membrane	transport - amino acid
ERG5	orf19.5178	-1.021	C-22 sterol desaturase ERG5	endoplasmic reticulum	metabolism - ergosterol biosynthesis
TCC1	orf19.6734	-1.007	Tcc1p	nucleus	stress response to pH, morphogenesis
ACB1	orf19.7043.1	-1.005	Long-chain fatty acid transporter	cytoplasm	metabolism - fatty acid biosynthesis
PGI1	orf19.3888	-1.001	Glucose-6-phosphate isomerase	cytoplasm	metabolism - carbohydrate catalysis
TDH3	orf19.6814	-0.982	Glyceraldehyde-3-phosphate dehydrogenase	cytoplasm, cell wall, plasma membrane	metabolism - carbohydrate catalysis
Hypothetical	orf19.3053	-0.967	Uncharacterized protein	biofilm, extracellular*	unknown
ERG10	orf19.1591	-0.962	Acetyl-CoA acetyltransferase	cytoplasm	metabolism - ergosterol biosynthesis

PMT1	orf19.5171	-0.947	Dolichyl-phosphate-mannose--protein mannosyltransferase 1	endoplasmic reticulum	cell wall/biofilm formation
SWE1	orf19.4867	-0.931	Mitosis inhibitor protein kinase SWE1	nucleus	cell cycle, morphogenesis
PHO85	orf19.6846	-0.930	Cyclin-dependent serine/threonine-protein kinase	cytoplasm, nucleus, cell surface	cell cycle, morphogenesis
ACC1	orf19.7466	-0.926	Acetyl-CoA carboxylase	intracellular membranes	metabolism - lipid/fatty acid
CUP1	orf19.3940.1	-0.911	Cup1p	membrane	copper transport, copper homeostasis
CDC28	orf19.3856	-0.909	Cyclin-dependent kinase 1	endoplasmic reticulum	cell cycle, morphogenesis
CWH8	orf19.3682	-0.894	Dolichyldiphosphatase	endoplasmic reticulum	protein modification
YVC1	orf19.2209	-0.894	Calcium channel YVC1	vacuole membrane	signal transduction, oxidative stress response
SUN41	orf19.3642	-0.874	Secreted beta-glucosidase SUN41	cell wall, biofilm matrix	cell cycle, morphogenesis
SLT11	orf19.5364	-0.869	Pre-mRNA-splicing factor SLT11	nucleus	mRNA processing
Hypothetical	orf19.5510	-0.856	WAC domain-containing protein	nucleus	unknown
PHO84	orf19.655	-0.839	Phosphate transporter	membrane	ion transport, stress response
SAM2	orf19.657	-0.828	S-adenosylmethionine synthase	cell wall, plasma membrane	metabolism - sulfur amino acid biosynthesis
DPP1	orf19.656	-0.822	Dpp1p	membrane	metabolism - lipid/fatty acid biosynthesis
MTS1	orf19. 4831	-0.820	Sphingolipid C9-methyltransferase	membrane**	metabolism - lipid/fatty acid biosynthesis
CRP1	orf19.4784	-0.806	Crp1p	membrane	copper transport, copper homeostasis
ATO10	orf19.3263	-0.806	Ato10p	membrane**	transport

Supplemental table 2: Upregulated genes in *C. albicans* NSS response

Gene Name	ORF	log FC	Product	Localization	Biological Process
ALS1	orf19.5741	3.987	Adhesion protein	cell wall, plasma membrane	adhesion
YmL25	orf19.7675	3.300	ribosomal protein	mitochondria	mitochondria biosynthesis, metabolism
PCK1	orf19.7514	2.457	Phosphoenolpyruvate carboxykinase	cytoplasm	metabolism-glycolysis
AOX2	orf19.4773	2.255	alternate oxidase	mitochondria	Electron transport and ATP synthesis
Hypothetical	orf19.7077	2.105	Putative ferric chelate reductase	plasma membrane/membranes	transport/ion transport/electron transport
SOU1	orf19.2896	1.651	Sorbose reductase	mitochondria	metabolism - carbohydrate
CTR1	orf19.3646	1.587	Copper transport protein	cell membrane, plasma membrane	transport/ion transport
Hypothetical	orf19.670.2	1.438	unknown function	unknown	unknown
MAE1	orf19.3419	1.436	Malate dehydrogenase	cytosol/mitochondria	metabolism - cellular amino acid, pyruvate
FUM12	orf19.6724	1.415	Fumarate hydratase	cytosol/mitochondria/nucleus	metabolism - malate
FRE7	orf19.6139	1.388	iron transporter	membranes	transport/ion transport/electron transport
Hypothetical	orf19.6090	1.387	ribosomal protein	nucleus	unknown
Hypothetical	orf19.2165	1.362	unknown function	unknown	unknown
YHM1	orf19.6854	1.360	Mitochondrial GTP/GDP carrier protein 1	membranes/mitochondria	transport - analytes
MIR1	orf19.4885	1.349	Mitochondrial phosphate carrier protein,	mitochondria	transport -ion
PAM18	orf19.4190	1.330	Mitochondrial import inner membrane translocase	mitochondria	transport - peptides
PET9	orf19.930	1.312	ADP/ATP translocase	mitochondria	transport - analytes
HPT1	orf19.5832	1.303	Hypoxanthine phosphoribosyltransferase	cell wall, biofilm matrix	nutrient acquisition
YmL24/YmL14	orf19.828	1.280	ribosomal protein	mitochondria	mitochondria biosynthesis and protein expression
IDH2	orf19.5791	1.275	mitochondrial Isocitrate dehydrogenase	mitochondria	metabolism - TCA cycle
TRR1	orf19.4290	1.258	Thioredoxin reductase	cytosol, biofilm matrix	oxidative stress response

SDS24	orf19.5118	1.253	Protein SDS23	cytoplasm, nucleus	cell cycle, DNA replication and cell separation
FPR3	orf19.1030	1.252	FK506-binding protein 3	nucleus	protein folding
IDH1	orf19.4826	1.224	mitochondrial Isocitrate dehydrogenase	mitochondria	metabolism - TCA cycle
Hypothetical	orf19.698	1.223	Uncharacterized protein	endoplasmic reticulum	unknown
FET34	orf19.4215	1.211	Ferroxidase	plasma membrane, extracellular matrix	transport, ion transport, iron homeostasis
FCA1	orf19.4195.1	1.206	Cytosine deaminase	cytoplasm, nucleus	metabolism - cytosine and UMP synthesis
YmL11	orf19.3797	1.204	Mitochondrial 54S ribosomal protein YmL11	mitochondria	mitochondrial biosynthesis and protein expression
YNK1	orf19.4311	1.188	Nucleoside diphosphate kinase	cytoplasm, extracellular	metabolism - nucleotide
RSM7	orf19.4018	1.183	Mitochondrial 37S ribosomal protein RSM7	mitochondria	mitochondrial biosynthesis and protein expression
FTR2	orf19.7213	1.183	Ftr2p - iron transporter	membrane	transporter, ion transporter, metal homeostasis
karyopherin	orf19.2489	1.176	Putative karyopherin beta	cytoplasm	nuclear import
MRPL10	orf19.3532	1.153	Mitochondrial 54S ribosomal protein YmL10/YmL18	mitochondria	mitochondrial biosynthesis and protein expression
GUA1	orf19.4813	1.151	GMP synthase	cytoplasm	metabolism - glutamine biosynthesis
KGD2	orf19.6126	1.113	Dihydrolipoyllysine-residue succinyltransferase	mitochondria	metabolism - TCA cycle
MRPS9	orf19.5230	1.113	mitochondrial 37S ribosomal protein S9	mitochondria	mitochondrial biosynthesis and protein expression
TUF1	orf19.6047	1.110	Elongation factor Tu	mitochondria	mitochondrial biosynthesis and protein expression
KGD1	orf19.6165	1.109	Oxoglutarate dehydrogenase	mitochondria	metabolism - TCA cycle
Hypothetical	orf19.93	1.103	Uncharacterized protein	mitochondria, nucleus	unknown

URA2	orf19.2360	1.101	Bifunctional carbamoylphosphate synthetase/aspartate transcarbamylase	cytoplasm	metabolism - pyrimidine, glutamine biosynthesis
MAM33	orf19.7187	1.100	Mam33p	mitochondria	mitochondrial biosynthesis and protein expression
Hypothetical	orf19.5517	1.097	predicted NADP-dependent alcohol dehydrogenase	cytoplasm	unknown
TIM23	orf19.1361	1.095	Mitochondrial import inner membrane translocase subunit TIM23	mitochondria	mitochondrial biosynthesis and protein expression
CRG1	orf19.633	1.092	Crg1p	cytoplasm	metabolism - lipid homeostasis
TIM9	orf19.6696	1.070	Mitochondrial import inner membrane translocase subunit TIM9	mitochondria	mitochondrial biosynthesis and protein expression
GPX2	orf19.85	1.064	Glutathione peroxidase	cytoplasm, mitochondria	oxidative stress response
MRPL4	orf19.6136	1.058	Mitochondrial 54S ribosomal protein L4	mitochondria	mitochondrial biosynthesis and protein expression
Hypothetical	orf19.5114.1	1.045	unknown function	membrane**	unknown
SDH12	orf19.2871	1.038	Succinate dehydrogenase	mitochondria	metabolism - TCA cycle
YmL31	orf19.1485	1.025	Mitochondrial 54S ribosomal protein L31	mitochondria	mitochondria biosynthesis and protein expression
ATP1	orf19.6854	1.021	ATP synthase subunit alpha	mitochondria	Electron transport and ATP synthesis
COI1	orf19.5063	1.020	Coi1p	extracellular	biofilm
RAD16	orf19.2969	1.020	DNA repair protein	nucleus	DNA repair/genomic stability
Hypothetical	orf19.1862	1.020	SBDS domain-containing protein	biofilm matrix	unknown
ATP4	orf19. 3579	1.016	ATP synthase subunit 4	mitochondria	Electron transport and ATP synthesis
Hypothetical	orf19.4450.1	1.015	Uncharacterized protein	membrane**	unknown
Hypothetical	orf19.35	1.010	Protein kinase domain-containing protein	unknown	unknown

YmL35	orf19.863	0.996	Mitochondrial 54S ribosomal protein YmL35	mitochondria	mitochondria biosynthesis and protein expression
MRPS17	orf19.4176	0.988	Mitochondrial 37S ribosomal protein MRPS17	mitochondria	mitochondria biosynthesis and protein expression
Hypothetical	orf19.2048	0.982	unknown function	membrane	unknown
YHB1	orf19.3707	0.979	Flavohemoprotein	cytoplasm	nitrosative stress response
SDH2	orf19.637	0.972	Succinate dehydrogenase iron-sulfur subunit	mitochondria	metabolism - TCA cycle
TOM40	orf19.6524	0.966	Tom40p	mitochondria	mitochondria biosynthesis
MRPL19	orf19.6231	0.965	Mitochondrial 54S ribosomal protein YmL19	mitochondria	mitochondria biosynthesis and protein expression
YmL23	orf19.3348	0.959	Mitochondrial 54S ribosomal protein L23	mitochondria	mitochondrial biosynthesis and protein expression
YMR31	orf19.4225.1	0.952	Mitochondrial 37S ribosomal protein YMR31	mitochondria	mitochondrial biosynthesis and protein expression
Hypothetical	orf19.915	0.945	Uncharacterized protein	unknown	unknown
RIP1	orf19.5893	0.944	Cytochrome b-c1 complex subunit Rieske, mitochondrial	mitochondria	Electron transport and ATP synthesis
YmL32	orf19.549	0.936	Mitochondrial 54S ribosomal protein YmL32	mitochondria	mitochondria biosynthesis and protein expression
Hypothetical	orf19.7215.3	0.931	Uncharacterized protein	mitochondria	unknown
RSM27	orf19.3297	0.925	Mitochondrial 37S ribosomal protein RSM27	mitochondria	mitochondria biosynthesis and protein expression
RML2	orf19.5420	0.919	Mitochondrial 54S ribosomal protein RML2	mitochondria	mitochondria biosynthesis and protein expression
CYC1	orf19.1770	0.917	Cytochrome c	mitochondria	Electron transport and ATP synthesis
Hypothetical	orf19.2275	0.913	Putative Mitochondrial nucleoid protein	mitochondria	unknown

YmL9	orf19.7485	0.911	Mitochondrial 54S ribosomal protein YmL9	mitochondria	mitochondria biosynthesis and protein expression
MGE1	orf19.2524	0.909	GrpE protein homolog	mitochondria	mitochondria biosynthesis and protein expression
Hypothetical	orf19.2414	0.893	Mpm1 Ortholog	mitochondria	unknown
SPE3	orf19.2250	0.886	Spermidine synthase	cytoplasm	metabolism - polyamine biosynthesis
YmL22	orf19.3367	0.884	Mitochondrial 54S ribosomal protein YmL22	mitochondria	mitochondria biosynthesis and protein expression
YmL17/YmL30	orf19.585	0.877	Mitochondrial 54S ribosomal protein YmL17/YmL30	mitochondria	mitochondria biosynthesis and protein expression
HSP60	orf19.717	0.861	Mitochondrial Heat shock protein 60	mitochondria	mitochondria biosynthesis and protein expression; stress response
ATP5	orf19.5419	0.860	Mitochondrial ATP synthase subunit 5	mitochondria	Electron transport and ATP synthesis
CBF5	orf19.1833	0.859	H/ACA ribonucleoprotein complex subunit CBF5	nucleus	RNA processing
Hypothetical	orf19.6492	0.859	Protein kinase domain-containing protein	unknown	unknown
ATP20	orf19.3757	0.849	Mitochondrial ATP synthase subunit 20	mitochondria	Electron transport and ATP synthesis
MRPS35	orf19.3559	0.841	Mitochondrial 37S ribosomal protein MRPS35	mitochondria	mitochondria biosynthesis and protein expression
SSC1	orf19.1896	0.838	Mitochondrial Heat shock protein SSC1	mitochondria	mitochondria biosynthesis and protein expression; stress response
Hypothetical	orf19.7621	0.837	Uncharacterized protein	nucleus*	unknown
QCR2	orf19.2644	0.836	Mitochondrial Cytochrome b-c1 complex subunit 2	mitochondria	Electron transport and ATP synthesis
MDN1	orf19.4697	0.833	Midasin	nucleus	nuclear import

ACO1	orf19.6385	0.832	Mitochondrial Aconitate hydratase	mitochondria	metabolism - TCA cycle
CAALFM_CR01 370CA	orf19.2520	0.826	Mitochondrial 37S ribosomal protein MRPS28	mitochondria	mitochondria biosynthesis and protein expression
Hypothetical	orf19.6853	0.820	Uncharacterized protein	unknown	unknown
ATP14	orf19.5491. 1	0.819	Mitochondrial ATP synthase subunit 14	mitochondria	Electron transport and ATP synthesis
RSM23	orf19.3480	0.811	Mitochondrial 37S ribosomal protein RSM23	mitochondria	mitochondria biosynthesis and protein expression
hypothetical	orf19.760	0.810	Mitochondrial Ribosomal protein	mitochondria	unknown
ASR1	orf19.2344	0.808	Asr1p	unknown	stress response
TSA1	orf19.7417	0.806	Peroxiredoxin TSA1-A	cytoplasm, nucleus, cell surface	oxidative stress response
SHA3/SKS1	orf19. 3669	0.806	Putative serine/threonine protein kinase	cytoplasm	signal transduction
MDH1-1	orf19.4602	0.801	Malate dehydrogenase	mitochondria	metabolism - TCA cycle

Supplemental table 3: Downregulated genes in *C. albicans* Mechanical Shear response

Gene	ORF	log2FC	Product	Localization	Biological Process
ASC2	orf19.1064)	-5.99	Acetyl-coenzyme A synthetase 2	cytoplasm	Metabolism -
CPR6	orf19.7654	-3.77	Putative peptidyl-prolyl cis-trans isomerase	cytoplasm	stress response - protein folding
hypothetical	orf19.449	-1.73	uncharacterized protein	mitochondrion	metabolism - phosolipid biosynthesis
HSP21	orf19.822	-1.46	Small heat shock protein 21	cell surface	stress response
SNZ1	orf19.2947	-1.42	Pyridoxal 5'-phosphate synthase	cytoplasm	metabolism - vitamin B synthesis
hypothetical	orf19.33	-1.33	uncharacterized protein	unknown	unknown
PLB1	orf19.689)	-1.32	Lysophospholipase 1	extracellular	metabolism - nutrient procurement
YCG1	orf19.1622	-1.21	Condensin subunit	nucleus - chromosomes	cell-division
hypothetical	orf19.411	-1.17	Uncharacterized protein	unknown	unknown
YHB1	orf19.3707	-1.03	Flavohemoprotein	cytoplasm	stress response - nitrosative
ECM21	orf19.4887	-1.02	Ecm21p	cytoplasm	transport - protein
MSH6	orf19.4945	-1.00	DNA mismatch repair protein	nucleus	DNA replication and repair
HEM13	orf19.2803	-1.00	Coproporphyrinogen oxidase	extracellular matrix, mitochondria, cytoplasm	metabolism - protoporphyrin biosynthesis
PSA2	orf19.4943	-0.98	Psa2p - nucleotidyltransferase activity	cytoplasm	metabolism - cellular nitrogen compound biosynthesis
hypothetical	orf19.1691	-0.95	HIG1 domain-containing protein	mitochondrion	electron transport/respiration
SSA1	orf19.4980	-0.95	Heat shock protein SSA1	cytoplasm, cell wall	stress response, protein folding
SNO1	orf19.2948	-0.94	Glutaminase	cytoplasm	metabolism - glutamine
hypothetical	orf19.2001	-0.94	Fe2OG dioxygenase domain-containing protein	endoplasmic reticulum	metabolism
HNM4	orf19.2946	-0.93	Hnm4p	plasma membrane	transport
hypothetical	orf19.787.1	-0.93	unknown	unknown	unknown
hypothetical	orf19.1785	-0.93	PI31_Prot_C domain-containing protein	cytoplasm	unknown

hypothetical	orf19.3661	-0.92	Putative ubiquitin-specific protease	cytoplasm/nucleus	protein degradation/regulation
hypothetical	orf19.3051	-0.92	TLC domain-containing protein	membrane	lipid homeostasis
hypothetical	orf19.5813	-0.91	Ubiquitin-like domain-containing protein	cytoplasm	protein degradation/regulation
XYL2	orf19.7676	-0.90	L-iditol 2-dehydrogenase	extracellular, cell wall	metabolism - sorbitol catalysis
hypothetical	orf19.3302	-0.89	CBM21 domain-containing protein	cytoplasm	metabolism - regulation of glycogen
hypothetical	orf19.1785	-0.89	PI31_Prot_C domain-containing protein	cytoplasm	unknown
CAS1	orf19.1135	-0.89	DNA helicase	nucleus	DNA replication and repair
hypothetical	orf19.1310	-0.88	unknown	unknown	unknown
HSP78	orf19.882	-0.88	Heat shock protein 78, mitochondrial	mitochondrion	stress response
GCV1	orf19.5519	-0.88	Aminomethyltransfera se	mitochondrion	metabolism - glycine catalysis
NAT4	orf19.4664	-0.88	N-alpha-acetyltransferase 40	cytoplasm, nucleus	regulation, chromatin - histone modification
LYS22	orf19.4506	-0.87	Homocitrate synthase	unknown	metabolism
hypothetical	orf19.3051	-0.87	unknown	unknown	unknown
HSP104	orf19.6387	-0.87	Chaperone ATPase	cytoplasm	stress response- protein folding
CRZ2	orf19.2356	-0.86	Transcriptional regulator CRZ2	nucleus	transcriptional regulation - biofilm, adhesion
PTC4	orf19.6638	-0.85	Type 2C protein phosphatase	mitochondrion	regulation
ATG15	orf19.10915	-0.84	Putative lipase ATG15	endosome	autophagy
RNR22	orf19.1868	-0.83	Rnr22p	membrane	metabolism - deoxyribonucleotide biosynthetic process
PRD1	orf19.434	-0.82	Metalloendopeptidase	mitochondrion	protein degradation/regulation
ZRT2	orf19.1585	-0.81	Low-affinity Zn(2+) transporter	membrane	transport
HSP104	orf19.6387	-0.81	Chaperone ATPase	cytoplasm	stress response- protein folding
DAP1	orf19.489	-0.81	Dap1p	endomembranes	unknown
CDR4	orf19.5079	-0.81	ABC transporter CDR4	membranes	transport
STF2	orf19.2107.1	-0.80	Stf2p	unknown	unknown
hypothetical	orf19.951	-0.80	Uncharacterized protein	unknown	Unknown

Supplemental table 4: Upregulated genes in *C. albicans* Mechanical Shear response

Gene name	ORF	LogFC	Product	Subcellular localization	Biological process
hypothetical	orf19.5049	2.30	Uncharacterized protein	unknown	unknown
SCW11	orf19.3893	2.27	Putative glucan endo-1\3-beta-D-glucosidase	cell wall	metabolism - nutrient procurement
MAK16	orf19.5500	2.25	Protein MAK16	nucleus	RNA processing
hypothetical	orf19.7197	2.11	Nucleolar complex-associated protein 3	nucleus	DNA replication and ribosome biogenesis
FGR41	orf19.4910	2.11	Filamentous growth regulator 41	extracellular	adhesion, morphogenesis, and filamentous growth
DBP2	orf19.171	2.05	ATP-dependent RNA helicase DBP2	nucleus	transcription
REI1	orf19.59	1.97	Rei1p	cytoplasm	unknown
RRT14	orf19.1708	1.94	Regulator of rDNA transcription 14	nucleus	transcription
SPB1	orf19.76	1.89	AdoMet-dependent rRNA methyltransferase SPB1	nucleus - nucleolus	ribosome biogenesis
PGA38	orf19.2758	1.88	Pga38p	cell wall	adhesion, morphogenesis and filamentous growth
CHR1	orf19.11240	1.84	ATP-dependent RNA helicase CHR1	nucleus	ribosome biogenesis
hypothetical	orf19.2319	1.82	NUC153 domain-containing protein	nucleus	ribosome biogenesis
NA	orf19.2386	1.82	U3 small nucleolar RNA-associated protein 11	nucleus	ribosome biogenesis
RRP15	orf19.563	1.82	RRP15	ribosome	ribosome biogenesis
CIC1	orf19.124	1.80	Cic1p	ribosome	ribosome biogenesis
hypothetical	orf19.2934	1.79	C2H2-type domain-containing protein	nucleus, cytoplasm	ribosome biogenesis
CHT3	orf19.7586	1.78	Chitinase 3	cell wall	cell wall biogenesis
RRP8	orf19.3630	1.77	Ribosomal RNA-processing protein 8	nucleus	ribosome biogenesis
ASH1	orf19.12803	1.76	Transcriptional regulatory protein ASH1	nucleus	transcriptional regulations
hypothetical	orf19.6886)	1.75	Ribosome biogenesis protein NOP53	nucleus	ribosome biogenesis
hypothetical	orf19.2167	1.73	Ribosome biosynthesis protein	nucleus	ribosome biogenesis
NOP6	orf19.6236	1.71	Nop6p	nucleus	ribosome biogenesis

SAS10	orf19.2717	1.71	rRNA-processing protein	nucleus	ribosome biogenesis
NOP14	orf19.5959	1.68	SnoRNA-binding rRNA-processing protein	nucleus	ribosome biogenesis
RPA12	orf19.2287	1.67	DNA-directed RNA polymerase subunit	nucleus	transcription
NA	orf19.2090	1.65	ATP-dependent RNA helicase	nucleus	ribosome biogenesis
SRP40	CAWG_01634	1.64	SRP40_C domain-containing protein	unknown	unknown
HBR3	orf19.6955	1.63	20S-pre-rRNA D-site endonuclease NOB1	nucleus	ribosome biogenesis
RIX1	orf19.6862	1.62	Pre-rRNA-processing protein RIX1	nucleus	ribosome biogenesis
NOP15	orf19.7050	1.60	rRNA-binding ribosome biosynthesis prote	nucleus	ribosome biogenesis
hypothetical	orf19.7104	1.60	Uncharacterized protein	cell wall	unknown - possibly adhesion
ECM1	orf19.5299	1.58	Ecm1p	nucleus	ribosome biogenesis
NOP4	orf19.5198	1.56	mRNA-binding ribosome biosynthesis protein	ribosome	ribosome biogenesis
hypothetical	orf19.4273	1.56	Uncharacterized protein	mitochondrion	unknown
hypothetical	orf19.4492	1.56	Uncharacterized protein	nucleus	ribosome biogenesis
HCA4	CaO19.10227	1.54	ATP-dependent RNA helicase DBP4	nucleus	ribosome biogenesis
KRR1	orf19.661	1.53	KRR1 small subunit processome component	nucleus	ribosome biogenesis
DBP8	CaO19.13973	1.52	ATP-dependent RNA helicase DBP8	nucleus	ribosome biogenesis
SCH9	orf19.829	1.51	Serine/threonine-protein kinase SCH9	cytoplasm	signal transduction, morphogenesis and filamentous growth
hypothetical	orf19.813	1.50	Uncharacterized protein	unknown	unknown
TSR2	orf19.2998	1.50	Tsr2p	nucleus	ribosome biogenesis
hypothetical	orf19.1388	1.50	Nucleolar protein 16	nucleus	ribosome biogenesis
hypothetical	orf19.107	1.50	RNA helicase	nucleus	ribosome biogenesis
hypothetical	orf19.1609	1.49	Kri1_C domain-containing protein	nucleus	ribosome biogenesis
ALS1	CaO19.13163	1.49	Agglutinin-like protein 1	cell wall	adhesion, morphogenesis and filamentous growth

hypothetical	orf19.3978	1.48	rRNA-processing protein EFG1	nucleus	ribosome biogenesis
AMN1	CaO19.1507,	1.47	Antagonist of mitotic exit network protein 1	cytoplasm, nucleus	cell cycle and cell division
PUS7	orf19.1753	1.47	Pseudouridine synthase	nucleus	ribosome biogenesis
hypothetical	orf19.7552	1.46	Uncharacterized protein	nucleus	ribosome biogenesis
FAD3	orf19.4933	1.45	Fad3p	membrane	metabolism - fatty acid biosynthetic process
NOP8	orf19.1091	1.45	Nop8p	ribosome	ribosome biogenesis
CGR1	orf19.2314	1.45	rRNA-processing protein CGR1	nucleus	ribosome biogenesis
ENP1	orf19.5507	1.45	SnoRNA-binding rRNA-processing protein	nucleus, preribosome, cytoplasm	ribosome biogenesis
hypothetical	orf19.962	1.45	Uncharacterized protein	unknown	unknown
NIP7	orf19.3478	1.45	60S ribosome subunit biogenesis protein NIP7	nucleus	ribosome biogenesis
LOC1	orf19.1642	1.44	60S ribosomal subunit assembly/export protein LOC1	nucleus	ribosome biogenesis
hypothetical	orf19.1687	1.44	RNA helicase	nucleus	RNA processing
UTP18	orf19.7154	1.44	Utp18p	nucleus	ribosome biogenesis
hypothetical	orf19.5267	1.43	Uncharacterized protein	unknown	unknown
BUD22	orf19.3287	1.43	Bud22p	nucleus	ribosome biogenesis
hypothetical	orf19.5019	1.42	Uncharacterized protein	unknown	unknown
RLP24	orf19.4191	1.42	Ribosome biogenesis protein RLP24	nucleus	ribosome biogenesis
PGA48	CaO19.6321	1.42	Cell wall protein PGA48	cell wall	cell wall stress
NOP12	orf19.809	1.42	Nucleolar protein 12	nucleus	ribosome biogenesis
NOP9	orf19.4479	1.41	Nucleolar protein 9	nucleus	ribosome biogenesis
BMS1	orf19.2504	1.39	GTPase	nucleus	ribosome biogenesis
RRP36	orf19.2362	1.39	rRNA biogenesis protein RRP36	nucleus	ribosome biogenesis
SRR1	CaO19.13265	1.38	Stress response regulator protein 1		stress response, morphogenesis
DIP2	orf19.5106	1.38	SnoRNA-binding rRNA-processing protein	nucleus	ribosome biogenesis

hypothetical	orf19.2917	1.36	Genetic interactor of prohibitins 3, mitochondrial	mitochondrion	ribosome biogenesis
MSS116	CaO19.12201	1.36	ATP-dependent RNA helicase MSS116, mitochondrial	mitochondrion	ribosome biogenesis
DBP3	CaO19.12334	1.36	ATP-dependent RNA helicase DBP3	nucleus	ribosome biogenesis
SDA1	orf19.6648	1.36	Protein SDA1	nucleus	ribosome biogenesis
NOC2	orf19.5850	1.36	mRNA-binding ribosome synthesis protein	nucleus	ribosome biogenesis
SSF1	orf19.6589	1.35	rRNA-binding ribosome biosynthesis protein	nucleus	ribosome biogenesis
RRP9	orf19.2830	1.34	Rrp9p	nucleus	ribosome biogenesis
BFR2	orf19.7624	1.34	Protein BFR2	nucleus	ribosome biogenesis
hypothetical	orf19.5067	1.33	Exosome complex protein	nucleus	gene regulation
ENP2	orf19.6686	1.33	Ribosome biosynthesis protein	nucleus	ribosome biogenesis
DBP7	CaJ7.0136,	1.33	ATP-dependent RNA helicase DBP7	nucleus	ribosome biogenesis
JIP5	CaO19.12208	1.32	WD repeat-containing protein JIP5	nucleus	ribosome biogenesis
DBP9	orf19.3393	1.30	ATP-dependent RNA helicase DBP9	nucleus	ribosome biogenesis
DBP10	orf19.5991	1.30	ATP-dependent RNA helicase DBP10	nucleus	ribosome biogenesis
FPR3	orf19.1030	1.30	FK506-binding protein 3	nucleus	protein folding
CAALFM_CR01950WA	orf19.2594	1.29	DNA-directed RNA polymerase I subunit	nucleus	transcription
YTM1	CaO19.12278	1.28	Ribosome biogenesis protein YTM1	nucleus	ribosome biogenesis
NA	orf19.6355	1.28	Ribosome biosynthesis protein	nucleus	ribosome biogenesis
hypothetical	orf19.4793	1.28	Uncharacterized protein	unknown	unknown
NSA2	CaO19.7424	1.28	Ribosome biogenesis protein NSA2	nucleus	ribosome biogenesis
snRNA		1.28	H/ACA box small nucleolar RNA (snoRNA)	nucleus	ribosome biogenesis
IMP4	orf19.603	1.27	SnoRNA-binding rRNA-processing protein	nucleus	ribosome biogenesis

MPP10	orf19.1915	1.26	U3 small nucleolar ribonucleoprotein protein MPP10	nucleus	ribosome biogenesis
NOG1	orf19.7384	1.26	Nucleolar GTP-binding protein 1	nucleus	ribosome biogenesis
NOP5	CaO19.1199	1.26	Nucleolar protein 58	nucleus	ribosome biogenesis
CSI2	orf19.5232	1.25	Csi2p	nucleus	ribosome biogenesis
RSM22	orf19.414	1.25	Mitochondrial 37S ribosomal protein RSM22	nucleus	ribosome biogenesis
BUD21	orf19.5430	1.25	Bud21p	nucleus	ribosome biogenesis
CRH11	CaO19.10221	1.25	Extracellular glycosidase CRH11	cell wall	cell wall biogenesis
NMD3	orf19.706	1.25	60S ribosomal export protein NMD3	nucleus, cytoplasm	ribosome biogenesis
MAK21	orf19.5912	1.25	RNA-binding ribosome biosynthesis protein	nucleus	ribosome biogenesis
NOP7	CaO19.11574	1.24	Pescadillo homolog	nucleus	ribosome biogenesis
RPA34	orf19.4896	1.24	DNA-directed RNA polymerase I subunit	nucleus	ribosome biogenesis
ENG1	CaO19.10584	1.23	Endo-1,3(4)-beta-glucanase 1	cell wall	metabolism, cell division
CAALFM_C306160CA	orf19.7397.1	1.23	Ribosome biogenesis protein C3_06160C_A	nucleus	ribosome biogenesis
hypothetical	orf19.6297	1.23	Pseudouridine synthase	cytoplasm	tRNA processing
ARX1	CaO19.10533	1.23	Probable metalloprotease ARX1	nucleus, cytoplasm	ribosome biogenesis
hypothetical	orf19.3463	1.22	Genetic interactor of prohibitins 3, mitochondrial	mitochondrion	ribosome biogenesis
RBE1	CaO19.7218	1.22	Repressed by EFG1 protein 1	cell wall	cell wall biogenesis
hypothetical	orf19.1772	1.22	Mitochondrial group I intron splicing factor CCM1	mitochondrion	RNA processing
hypothetical	orf19.2527	1.21	Uncharacterized protein	cytoplasm	unknown
SPB4	CaO19.11631	1.21	ATP-dependent rRNA helicase SPB4	nucleus	ribosome biogenesis
PXR1	orf19.3831	1.21	Protein PXR1	nucleus	ribosome biogenesis
RRS1	orf19.6014	1.20	Ribosome biogenesis regulatory protein	nucleus	ribosome biogenesis
DRS1	CaO19.7635	1.19	ATP-dependent RNA helicase DRS1	nucleus	ribosome biogenesis

RPF2	orf19.3553	1.19	Ribosome production factor 2 homolog	nucleus	ribosome biogenesis
ALB1	orf19.7107	1.19	Ribosome biogenesis protein ALB1	nucleus	ribosome biogenesis
RPL7	orf19.3867	1.19	Rpl7p	nucleus	ribosome biogenesis
RRP6	orf19.58	1.18	Exosome nuclease subunit	nucleus	RNA processing
hypothetical	orf19.3547	1.18	PUM-HD domain-containing protein	nucleus	ribosome biogenesis
FRE10	orf19.1415	1.17	Fre10p	membrane	iron homeostasis
hypothetical	orf19.3470	1.17	tRNA 4-demethylwyosine synthase (AdoMet-dependent)	membrane	tRNA processing
UTP4	orf19.1633	1.16	Utp4p	nucleus	ribosome biogenesis
TOP1		1.16	DNA topoisomerase 1	nucleus, cytosol	DNA replication
hypothetical	orf19.7215	1.16	Uncharacterized protein	mitochondrion	unknown
rRNA-binding ribosome biosynthesis protein	orf19.3724	1.16	rRNA-binding ribosome biosynthesis protein	nucleus	ribosome biogenesis
hypothetical	orf19.6175	1.16	Fcf2 domain-containing protein	nucleus	RNA processing
hypothetical	orf19.6090	1.16	Uncharacterized protein	nucleus	unknown
hypothetical	orf19.7011	1.16	mRNA-binding protein	nucleus	RNA processing
snR73	-	1.15	(snR73) C/D box small nucleolar RNA (snoRNA)	nucleus	ribosome biogenesis
SOF1	orf19.5407	1.14	rRNA-processing protein	nucleus	ribosome biogenesis
PWP1	orf19.4640	1.14	rRNA-processing protein	nucleus	ribosome biogenesis
MRD1	orf19.1646	1.14	Multiple RNA-binding domain-containing protein 1	nucleus	RNA processing
ZPR1	orf19.3300	1.14	Zinc finger-containing protein	nucleus, cytoplasm	unknown
UTP13	orf19.4268	1.13	Utp13p	nucleus	ribosome biogenesis
CAALFM_CR05550CA	orf19.5847	1.13	DNA-directed RNA polymerase subunit beta	nucleus	transcription
NA	orf19.1578	1.12	Uncharacterized protein	nucleus	ribosome biogenesis
SIK1	orf19.7569	1.12	snoRNP complex protein	nucleus	ribosome biogenesis

PWP2	orf19.3276	1.11	SnoRNA-binding rRNA-processing protein	nucleus	ribosome biogenesis
MAK5	CaO19.11024	1.10	ATP-dependent RNA helicase MAK5	nucleus	ribosome biogenesis
hypothetical	orf19.2669	1.09	Integrase catalytic domain-containing protein	cytoplasm, nucleus	unknown
hypothetical	orf19.5835	1.09	Uncharacterized protein	nucleus	ribosome biogenesis
hypothetical	orf19.1791	1.09	Uncharacterized protein	unknown	unknown
PUS1	orf19.3477	1.08	tRNA pseudouridine synthase 1	nucleus	RNA processing
hypothetical	orf19.2631	1.08	Elongator subunit	cytoplasm	tRNA processing
CAALFM_CR10750CA	orf19.7664	1.08	zf-LYAR domain-containing protein	nucleus	ribosome biogenesis
hypothetical	orf19.1893	1.07	Uncharacterized protein	unknown	unknown
MRT4	orf19.5550	1.07	Ribosome assembly factor mrt4	nucleus	ribosome biogenesis
FLU1	orf19.6577	1.07	Major facilitator superfamily multidrug transporter FLU1	plasma membrane	transport
ELF1	orf19.7332	1.06	Elf1p	nucleus, plasma membrance, cytoplasm	ribosome biogenesis
ERB1	CaO19.1047	1.06	Ribosome biogenesis protein ERB1	nucleus	ribosome biogenesis
FAL1	CaO19.10024	1.06	ATP-dependent RNA helicase FAL1	nucleus	ribosome biogenesis
hypothetical	orf19.1697	1.05	Uncharacterized protein	cytoplasm	unknown
RPA190	orf19.1839	1.04	DNA-directed RNA polymerase subunit	nucleus	transcription
TIM23	orf19.1361	1.04	Mitochondrial import inner membrane translocase subunit TIM23	mitochondrion	transport - peptide
NAT10	orf19.512	1.03	RNA cytidine acetyltransferase	nucleus	RNA processing
hypothetical	orf19.7422	1.03	Uncharacterized protein	nucleus	unknown
CAALFM_CR04170WA	orf19.501	1.03	rRNA (Cytosine-C5-)-methyltransferase	nucleus	ribosome biogenesis
MIS12	orf19.7534	1.01	Mis12p	cytoplasm	metabolism - carbon
RIO2	orf19.6369	1.00	Non-specific serine/threonine protein kinase	cytoplasm, nucleus	morphogensis, nuclear RNA import

hypothetical	orf19.4029	1.00	Uncharacterized protein	cytoplasm	unknown
NOP1	orf19.3138	1.00	rRNA 2'-O-methyltransferase fibrillarin	nucleus	ribosome biogenesis
UTP22	orf19.1569	0.99	U3 small nucleolar RNA-associated protein 22	nucleus	ribosome biogenesis
hypothetical	orf19.7160	0.97	Uncharacterized protein	unknown	unknown
TRM1	orf19.3265	0.97	tRNA (guanine(26)-N(2))-dimethyltransferase	mitochondrion	RNA processing
HAS1	CaO19.11444	0.96	ATP-dependent RNA helicase HAS1	nucleus	ribosome biogenesis
RPO41	orf19.6041	0.93	DNA-directed RNA polymerase	mitochondrion	Mitochondrial Genome maintence
NRI1	orf19.1441	0.93	Uncharacterized protein	nucleus	transcriptional regulation
GUA1	CaO19.12276	0.92	GMP synthase [glutamine-hydrolyzing]	cytoplasm	metabolism - glutamine
MDN1	orf19.4697	0.91	Midasin	nucleus	ribosome biogenesis
DUS3	orf19.1565	0.91	tRNA-dihydrouridine(47) synthase [NAD(P)(+)]	cytoplasm, nucleus	RNA processing
hypothetical	orf19.5342.2	0.89	Uncharacterized protein	unknown	unknown
SNU13	orf19.5885	0.87	13 kDa ribonucleoprotein-associated protein	nucleus	RNA processing, ribosome biogenesis
GUT2	orf19.3133	0.86	Glycerol-3-phosphate dehydrogenase	cytoplasm	metabolism
hypothetical	orf19.4176	0.86	Mitochondrial 37S ribosomal protein MRPS17	nucleus	ribosome biogenesis
RPA135	orf19.7062	0.86	DNA-directed RNA polymerase subunit beta	nucleus	transcription
TSR1	orf19.6417	0.86	Tsr1p	nucleus	ribosome biogenesis
hypothetical	orf19.4532	0.84	Uncharacterized protein	nucleus, cytoplasm	regualtion of respiration
GAR1	orf19.1164	0.83	H/ACA ribonucleoprotein complex subunit	nucleus	ribosome biogenesis
CBF5	orf19.1833	0.82	H/ACA ribonucleoprotein complex subunit CBF5	nucleus	ribosome biogenesis
hypothetical	orf19.3367	0.82	Mitochondrial 54S ribosomal protein YmL22	mitochondrion	mitochondrial biogenesis

SEH1	orf19.2186	0.82	Seh1p	membrane	transport
HMT1	orf19.3291	0.81	Protein-arginine omega-N methyltransferase	nucleus	protein modification
BFR2	orf19.7624	0.81	Protein BFR2	nucleus	ribosome biogenesis
hypothetical	orf19.3103	0.80	DNA-directed RNA polymerase subunit	nucleus	transcription

Supplemental Table 5: Downregulated genes in *C. albicans* reduced adhesion conditions

Gene	ORF	LogFC	Product	Subcellular Localization	Biological Process
hypothetical	orf19.33	-21.604	unknown	unknown	unknown
TDH3	orf19.6814	-14.081	NAD-linked glyceraldehyde-3-phosphate dehydrogenase	cell wall, biofilm matrix	adhesion to host cells
HRT2	orf19.4624	-13.866	phosphatase	unknown	unknown
CRP1	orf19.4784	-12.082	Copper transporter	plasma membrane	transport - copper, silver, cadmium
FAS2	orf19.5949	-11.731	Alpha subunit of fatty-acid synthase	cytosol	metabolism - fatty acid synthase
CUP1	orf19.3940.1	-10.629	Metallothionein	unknown	copper homeostasis
HSP21	orf19.822	-10.577	Small heat shock protein	cell surface	stress response
CDC19	orf19.3575	-10.564	Pyruvate kinase at yeast cell surface	cell wall, cell surface	morphogenesis, filamentous growth
FAS1	orf19.979	-9.656	Beta subunit of fatty-acid synthase	cytosol	metabolism - fatty acid biosynthesis
FBA1	orf19.4618	-9.525	Fructose-bisphosphate aldolase	cell wall	metabolism - carbohydrate
HEM13	orf19.2803	-8.834	Coproporphyrinogen III oxidase	cytosol, extracellular	metabolism - ergosterol and heme biosynthesis
HSP104	orf19.6387	-7.607	Heat-shock protein	cell surface	stress response
HSP70	orf19.4980	-7.526	hsp70 chaperone	cytoplasm, nucleus, cell wall	stress response
XYL2	orf19.7676	-7.164	D-xylulose reductase	cell wall- hyphal	proteolysis
PGA10	orf19.5674	-7.024	GPI anchored membrane protein	cell wall	metabolism and transport - iron homeostasis and ergosterol biosynthesis
hypothetical	orf19.7085	-6.958	unknown	unknown	unknown
HSP78	orf19.882	-6.808	Heat-shock protein	mitochondrial matrix	stress response
STF2	orf19.2107.1	-6.711	Protein involved in ATP biosynthesis	unknown	ATP biosynthetic process

GRP2	orf19.4309	-6.681	NAD(H)-linked methylglyoxal oxidoreductase involved in regulation of methylglyoxal and pyruvate levels	biofilm matrix	metabolism
PLB1	orf19.689	-6.429	Phospholipase B	extracellular matrix	metabolism - phospholipid catabolic process
CRZ2	orf19.2356	-5.659	C2H2 transcription factor	unknown	stress response
SNZ1	orf19.2947	-5.578	Stationary phase protein	cytoplasm, cell wall	metabolism
PFK26	orf19.4753	-5.568	Putative 6-phosphofructo-2-kinase	unknown	metabolism - glucose metabolism
hypothetical	orf19.5282	-5.567	unknown	unknown	unknown
hypothetical	orf19.449	-5.518	Putative phosphatidyl synthase	unknown	transport - carbohydrate
DDR48	orf19.4082	-5.393	unknown	unknown	unknown
hypothetical	orf19.3302	-4.971	unknown	unknown	unknown
GCV1	orf19.5519	-4.752	Putative T subunit of glycine decarboxylase	cytoplasm	metabolism - glycine degradation
DAP1	orf19.489	-4.730	Similar to mammalian membrane-associated progesterone receptors involved in DNA damage response	biofilm matrix	ergosterol biosynthesis
hypothetical	orf19.7531	-4.600	unknown	intracellular anatomical structure	drug transport
GDH2	orf19.2192	-4.543	Mitochondrial NAD-dependent glutamate dehydrogenase	cytosol	metabolism - glutamate catabolism
NA	orf19.5644	-4.437	membrane bending protein	cytoplasm	transport - carbohydrate; membrane formation
NA	orf19.4530.1	-4.257	unknown	unknown	unknown
NA	orf19.3051	-4.207	unknown	membrane	metabolism - ergosterol biosynthesis

PGA45	orf19.2451	-4.056	Putative GPI-anchored cell wall protein	cell wall	unknown
UPC2	orf19.391	-4.053	Zn2-Cys6 transcript factor	nucleus	transcription - ergosterol regulation
ECM331	orf19.4255	-3.965	GPI-anchored protein	cell wall, biofilm matrix	unknown
PLB4.5	orf19.1442	-3.923	Phospholipase B	extracellular matrix	metabolism - phospholipid catabolic process
hypothetical	orf19.7329	-3.882	ubiquitin-protein transferase	endoplasmic reticulum	ubiquitin dependent protein degradation
CHA1	orf19.1996	-3.832	catabolic ser/thr dehydratase	mitochondrial nucleoid	metabolism - L-serine catalysis
hypothetical	orf19.411	-3.829	unknown	unknown	unknown
hypothetical	orf19.1785	-3.809	unknown	unknown	unknown
hypothetical	orf19.3932	-3.802	RNA binding protein	unknown	transport - carbohydrate
RPR1	RPR11	-3.657	RNAse P RNA	ribonuclease complex	unknown
SNO1	orf19.2948	-3.471	Protein with a predicted role in pyridoxine metabolism	cytoplasm	metabolism - pyridoxine
hypothetical	orf19.5518	-3.448	unknown	unknown	unknown
hypothetical	orf19.3793	-3.446	unknown	unknown	unknown
ECM21	orf19.4887	-3.439	Predicted regulator of endocytosis of plasma membrane proteins	unknown	endocytosis
hypothetical	orf19.5620	-3.434	unknown	unknown	unknown
PSA2	orf19.4943	-3.408	Mannose-1-phosphate guanyl transferase	unknown	metabolism
hypothetical	orf19.4780	-3.374	Predicted MFS family membrane transporter	membrane	transport
NAT4	orf19.4664	-3.328	Putative histone acetyltransferase	unknown	phenotype switching
SWE1	orf19.4867	-3.271	kinase	nucleus	signal transduction, cell cycle
hypothetical	orf19.2826	-3.087	unknown	unknown	unknown
PGA7	orf19.5635	-3.049	GPI-linked hyphal surface antigen	cell surface	biofilm formation
hypothetical	orf19.3661	-3.003	Putative deubiquitinating enzyme	mitochondrial outer membrane	ubiquitin dependent protein degradation
hypothetical	orf19.36.1	-2.990	unknown	unknown	unknown

hypothetical	orf19.4735	-2.983	Ornithine cyclodeaminase family protein	unknown	unknown
hypothetical	orf19.6554	-2.966	unknown	unknown	unknown
LAP3	orf19.539	-2.959	Putative aminopeptidase	cytoplasm	transport - carbohydrate
hypothetical	orf19.3342	-2.854	unknown	unknown	ubiquitin dependent protein degradation
CZF1	orf19.3127	-2.776	Transcription factor	nucleus	signal transduction
hypothetical	orf19.3281	-2.764	histone H3-methyl-lysine-36 demethylase	unknown	transcriptional regulation
EBP1	orf19.125	-2.715	NADPH oxidoreductase	eisosome	eisosome assembly
NA	orf19.2515	-2.645	ZZ-type zinc finger protein	unknown	transport - carbohydrate
hypothetical	orf19.4280	-2.590	unknown	unknown	unknown
C2_02920W_A	orf19.5813	-2.589	putative adhesion molecule	unknown	unknown
hypothetical	orf19.3360	-2.578	unknown	unknown	unknown
hypothetical	orf19.7196	-2.575	vacuolar protease	vacuole	autophagy, protein degradation
hypothetical	orf19.7531	-2.560	unknown	unknown	unknown
hypothetical	orf19.2262	-2.521	putative quinone oxidoreductase	unknown	cell redox homeostasis
PEX22	orf19.1225	-2.517	Putative peroxin	unknown	unknown
hypothetical	orf19.1999	-2.494	unknown	unknown	unknown
RPN4	orf19.1069	-2.405	C2H2 transcription factor	nucleus	transcription, stress response
ATO2	orf19.2496	-2.328	unknown	membrane	transport - carbohydrate
MEP1	orf19.1614	-2.261	Ammonium permease	plasma membrane	transport - ammonium
unknown	orf19.1075	-2.199	unknown	unknown	unknown
hypothetical	orf19.1107	-2.195	unknown	cytoplasm	autophagy
ARO10	orf19.1847	-2.169	Aromatic decarboxylase	unknown	metabolism - aromatic amino acid catabolism
PUT2	orf19.3974	-2.140	Putative delta-1-pyrroline-5-carboxylate dehydrogenase	mitochondrion	metabolism - glutamate biosynthesis

Supplemental Table 6: Downregulated genes *C. albicans* in reduced adhesion conditions

Gene	ORF	LogFC	Product	Subcellular Localization	Biological Process
HHO1	orf19.5137.1	3.74	Histone H1	nucleus	transcriptional regulation
Yml25	orf19.7675	3.36	Mitochondrial 54S ribosomal protein YmL25	mitochondrion	mitochondrial biogenesis

RBT4	orf19.6202	2.75	Secreted protein RBT4	extracellular matrix	virulence
hypothetical	orf19.5049	2.38	uncharacterized protein	unknown	unknown
SCW11	orf19.3893	2.33	Putative glucan endo-1\3-beta-D-glucosidase	cell wall	cell wall biogenesis
DBP2	CaO19.171	2.32	ATP-dependent RNA helicase DBP2	nucleus, cytoplasm	ribosome biogenesis
RRP8	orf19.3630	2.26	Ribosomal RNA-processing protein 8	nucleus	ribosome biogenesis
MAK16	orf19.5500	2.23	Protein MAK16	nucleus	ribosome biogenesis
hypothetical	orf19.7160	2.22	uncharacterized protein	unknown	unknown
FGR41	CaO19.12376	2.09	Filamentous growth regulator 41	extracellular matrix	adhesion, virulence
CHR1	CaO19.11240	2.07	ATP-dependent RNA helicase CHR1	nucleus	ribosome biogenesis
hypothetical	orf19.4273	2.05	uncharacterized protein	mitochondrion	unknown
CAALFM_CR03360WA	orf19.2386	2.01	U3 small nucleolar RNA-associated protein 11	nucleus	rRNA processing
hypothetical	orf19.2934	2.00	C2H2-type domain-containing protein	nucleus, cytoplasm, ribosome	ribosome biogenesis
RRT14	orf19.1708	1.98	Regulator of rDNA transcription 14	nucleus	ribosome biogenesis
REI1	orf19.59	1.93	Rei1p	cytoplasm	ribosome biogenesis
AdoMet-dependent rRNA methyltransferase SPB1	orf19.76	1.89	AdoMet-dependent rRNA methyltransferase SPB1	nucleus	ribosome biogenesis
RRP15	orf19.563	1.85	Rrp15p	nucleus	ribosome biogenesis
NOP6	orf19.6236	1.82	Nop6p	nucleus	ribosome biogenesis
ASH1	CaO19.12803	1.78	Transcriptional regulatory protein ASH1	nucleus	transciptional regulation
hypothetical	orf19.2090	1.77	ATP-dependent RNA helicase	nucleus	ribosome biogenesis
MSS116	CaO19.12201	1.75	ATP-dependent RNA helicase MSS116, mitochondrial	mitochondrion	mitochondrial RNA Processing
PGA38	orf19.2758	1.74	Pga38p	cell wall	unknown
ECM1	orf19.5299	1.74	Ecm1p	nucleus, cytoplasm	ribosome biogenesis
TSR2	orf19.2998	1.74	Tsr2p	nucleus	rRNA processing
hypothetical	orf19.813	1.73	uncharacterized protein	unknown	unknown
SRR1	CaO19.13265	1.72	Stress response regulator protein 1	nucleus, cytoplasm, mitochondrion	stress response

RPA12	orf19.2287	1.72	DNA-directed RNA polymerase subunit	nucleus	transcription
CHT3	CaO19.7586	1.72	Chitinase 3	extracellular matrix, cell wall	cell wall biogenesis
CIC1	Cic1p	1.71	Cic1p	ribosome	ribosome biogenesis
hypothetical	orf19.5267	1.71	uncharacterized protein	unknown	unknown
hypothetical	orf19.2319	1.70	NUC153 domain-containing protein	nucleus	rRNA processing
hypothetical	orf19.2167	1.69	Ribosome biosynthesis protein	nucleus	ribosome biogenesis
NOP14	orf19.5959	1.69	SnoRNA-binding rRNA-processing protein	nucleus	ribosome biogenesis
hypothetical	orf19.107	1.69	RNA helicase	nucleus	RNA processing
NCE103	CaO19.1721	1.67	Carbonic anhydrase	cytoplasm, nucleus	virulence
SAS10	orf19.2717	1.67	rRNA-processing protein	nucleus	rRNA processing
RIX1	orf19.6862	1.65	Pre-rRNA-processing protein RIX1	nucleus	rRNA processing
CGR1	orf19.2314	1.65	rRNA-processing protein CGR1	nucleus	rRNA processing
ENP2	orf19.6686	1.64	Ribosome biosynthesis protein	nucleus	ribosome biogenesis
CAALFM_C504910WA	orf19.3978	1.63	rRNA-processing protein EFG1	nucleus	ribosome biogenesis
NOP16	orf19.1388	1.62	Nucleolar protein 16	nucleus	ribosome biogenesis
RSM22	orf19.414	1.61	Mitochondrial 37S ribosomal protein RSM22	mitochondrion	mitochondrial biogenesis
HBR3	orf19.6955	1.61	20S-pre-rRNA D-site endonuclease NOB1	nucleus	rRNA processing
NOP15	orf19.7050	1.60	rRNA-binding ribosome biosynthesis protein	nucleus	ribosome biogenesis
hypothetical	orf19.6886	1.59	Ribosome biogenesis protein NOP53	nucleus	ribosome biogenesis
hypothetical	orf19.6185	1.59	uncharacterized protein	unknown	unknown
DBP9	orf19.3393	1.58	ATP-dependent RNA helicase DBP9	nucleus	ribosome biogenesis, rRNA processing
AMN1	CaO19.1507	1.58	Antagonist of mitotic exit network protein 1	cytoplasm, nucleus	cell cycle, cell division
UTP18	orf19.7154	1.57	Utp18p	nucleus	rRNA processing
hypothetical	orf19.5905	1.56	Uncharacterized protein	unknown	unknown
KRR1	orf19.661	1.55	KRR1 small subunit processome component	nucleus	ribosome biogenesis

hypothetical	orf19.2320	1.54	Serine/threonine-protein kinase RIO1	cytoplasm	ribosome biogenesis
FAD3	orf19.4933	1.53	Fad3p	membrane	metabolism - lipid fatty acid biosynthesis
hypothetical	orf19.962	1.52	uncharacterized protein	unknown	unknown
CAWG_01634	CAWG_01634	1.52	SRP40_C domain-containing protein	nucleus	unknown
BUD22	orf19.3287	1.51	Bud22p	nucleus	rRNA processing
hypothetical	orf19.6297	1.49	Pseudouridine synthase	nucleus	RNA processing
LOC1	orf19.1642	1.47	60S ribosomal subunit assembly/export protein LOC1	nuclues	ribosome biogenesis
NOP12	orf19.809	1.47	Nucleolar protein 12	nucleus	ribosome biogenesis
NSA2	CaO19.7424	1.46	Ribosome biogenesis protein NSA2	nucleus	ribosome biogenesis
hypothetical	orf19.3470	1.46	tRNA 4-demethylwyosine synthase (AdoMet-dependent)	nucleus	tRNA processing
NOP9	orf19.4479	1.46	Nucleolar protein 9	nucleus	ribosome biogenesis
UTP4	orf19.1633	1.44	Utp4p	nucleus	ribosome biogenesis
NA	orf19.4793	1.43	Uncharacterized protein	nucleus	unknown
DSE1	CaO19.11112	1.43	DSE1	cell wall	cell wall biogenesis
hypothetical	orf19.1609	1.43	Kri1_C domain-containing protein	nucleus	ribosome biogenesis
HCA4	CaO19.10227	1.43	ATP-dependent RNA helicase DBP4	nucleus	ribosome biogenesis, rRNA processing
hypothetical	orf19.6175	1.43	Fcf2 domain-containing protein	nucleus	RNA processing
hypothetical	orf19.5019	1.42	uncharacterized protein	membrane	unknown
SOU1	CaO19.10414	1.42	Sorbose reductase SOU1	cytoplasm	metabolism - carbohydrate catalysis
CAALFM_C306160CA	orf19.7397.1	1.42	Ribosome biogenesis protein C3_06160C_A	nucleus	ribosome biogenesis
ENG1	CaO19.10584	1.41	Endo-1,3(4)-beta-glucanase 1	cell wall	cell wall biogenesis
snR31a	-	1.41	(snR31a) H/ACA box small nucleolar RNA (snoRNA)	nucleus	ribosome biogenesis
hypothetical	orf19.7104	1.41	uncharacterized protein	cell wall	unknown

RRP9	orf19.2830	1.41	RRP9	nucleus	ribosome biogenesis, rRNA processing
DBP8	CaO19.13973	1.41	ATP-dependent RNA helicase DBP8	nucleus	ribosome biogenesis, rRNA processing
BFR2	orf19.7624	1.40	Protein BFR2	nucleus	rRNA processing
CDC54	orf19.3761	1.40	DNA replication licensing factor MCM4	nucleus	cell cycle, cell division
PGA48	CaO19.6321	1.39	Cell wall protein PGA48	cell wall	cell wall biogenesis
NA	orf19.2362	1.39	rRNA biogenesis protein RRP36	nucleus	ribosome biogenesis, rRNA processing
SDA1	orf19.6648	1.39	Protein SDA1	nucleus	ribosome biogenesis
NOP4	orf19.5198	1.38	mRNA-binding ribosome biosynthesis protein	ribosome	ribosome biogenesis
CAALFM_CR01710WA	orf19.2564	1.38	uncharacterized protein	nucleus	ribosome biogenesis
NOP8	orf19.1091	1.38	Nucleolar protein 8	nucleus	ribosome biogenesis
TRM82	orf19.6477	1.38	tRNA (guanine-N(7)-)-methyltransferase non-catalytic subunit TRM82	nucleus	tRNA processing
hypothetical	orf19.1687	1.38	RNA helicase	nucleus	RNA processing
NIP7	orf19.3478	1.38	60S ribosome subunit biogenesis protein NIP7	nucleus	ribosome biogenesis
orf19.7552	orf19.7552	1.37	uncharacterized protein	nucleus	ribosome biogenesis, rRNA processing
SSF1	orf19.6589	1.35	ribosome biogenesis, rRNA processing	nucleus	ribosome biogenesis, rRNA processing
JIP5	CaO19.12208	1.34	WD repeat-containing protein JIP5	nucleus	ribosome biogenesis
TIM23	orf19.1361	1.34	Mitochondrial import inner membrane translocase subunit TIM23	mitochondrion	mitochondrial transport
ENP1	orf19.5507	1.33	SnoRNA-binding rRNA-processing protein	nucleus	rRNA processing
hypothetical	orf19.4492	1.33	uncharacterized protein	nucleus	ribosome biogenesis,

					rRNA processing
Yml11	orf19.3797	1.32	Mitochondrial 54S ribosomal protein YmL11	mitochondrion	mitochondrial biogenesis
AIM11	orf19.6156	1.32	Altered inheritance of mitochondria protein 11	membrane	cell division
PUS7	orf19.1753	1.32	Pseudouridine synthase	nucleus	metabolism - pseudouridine synthesis
RRS1	orf19.6014	1.32	Ribosome biogenesis regulatory protein	nucleus	ribosome biogenesis
RLP24	orf19.4191	1.31	Ribosome biogenesis protein RLP24	nucleus	ribosome biogenesis
hypothetical	orf19.4563	1.30	Putative methyltransferase	unknown	unknown
DBP7	CaJ7.0136	1.29	ATP-dependent RNA helicase DBP7	nucleus	ribosome biogenesis, rRNA processing
NOC2	orf19.5850	1.28	mRNA-binding ribosome synthesis protein	nucleus	ribosome biogenesis
RPC19	orf19.172	1.28	DNA-directed RNA polymerase core subunit	nucleus	transcription
PAM18	CaO19.11667	1.27	PAM18	mitochondrion	mitochondrial transport
hypothetical	orf19.2917	1.26	Genetic interactor of prohibitins 3, mitochondrial	mitochondrion	mitochondrial biogenesis
IMP4	orf19.603	1.26	SnoRNA-binding rRNA-processing protein	nucleus	rRNA processing
DIP2	orf19.5106	1.26	SnoRNA-binding rRNA-processing protein	nucleus	rRNA processing
hypothetical	orf19.5235	1.26	Mitochondrial 54S ribosomal protein YmL13	mitochondrion	mitochondrial biogenesis
DBP3	CaO19.12334	1.25	ATP-dependent RNA helicase DBP3	nucleus	ribosome biogenesis, rRNA processing
hypothetical	orf19.6355	1.23	Ribosome biosynthesis protein	nucleus	ribosome biogenesis
RCL1	orf19.1886	1.23	rRNA-processing endoribonuclease	nucleus	rRNA processing
PUS1	orf19.3477	1.23	tRNA pseudouridine synthase 1	nucleus	RNA processing
PXR1	orf19.3831	1.22	Protein PXR1	nucleus	ribosome biogenesis, rRNA processing
RPF1	orf19.2667	1.22	rRNA-binding ribosome biosynthesis protein	nucleus	ribosome biogenesis

BUD21	orf19.5430	1.21	Bud21p	nucleus	rRNA processing
MRD1	orf19.1646	1.21	Multiple RNA-binding domain-containing protein 1	nucleus	RNA processing
CSI2	orf19.5232	1.20	Csi2p	nucleus	ribosome biogenesis
BMS1	orf19.2504	1.20	BMS1	nucleus	ribosome biogenesis
PNO1	orf19.7618	1.20	Pre-rRNA-processing protein PNO1	cytoplasm, nucleus	ribosome biogenesis
ARX1	CaO19.10533	1.20	Probable metalloprotease ARX1	nucleus	ribosome biogenesis
SOF1	orf19.5407	1.19	rRNA-processing protein	cytoplasm, nucleus	ribosome biogenesis
hypothetical	orf19.1791	1.19	uncharacterized protein	unknown	unknown
NOP5	CaO19.1199	1.18	Nucleolar protein 58	nucleus	ribosome biogenesis, rRNA processing
NMD3	orf19.706	1.18	60S ribosomal export protein NMD3	nucleus	ribosome biogenesis
TOP1	-	1.18	DNA topoisomerase 1	nucleus, cytoplasm	DNA replication
hypothetical	orf19.7422	1.18	uncharacterized protein	nucleus	ribosome biogenesis
hypothetical	orf19.5835	1.18	uncharacterized protein	nucleus	ribosome biogenesis
MAK5	CaO19.11024	1.18	ATP-dependent RNA helicase MAK5	nucleus	ribosome biogenesis, rRNA processing
CAALFM_CR02420WA	orf19.3724	1.18	rRNA-binding ribosome biosynthesis protein	nucleus	ribosome biogenesis, rRNA processing
RPA34	orf19.4896	1.18	DNA-directed RNA polymerase I subunit	nucleus	transcription
hypothetical	orf19.5991	1.18	rRNA-binding ribosome biosynthesis protein	nucleus	ribosome biogenesis, rRNA processing
PCK1	orf19.7514	1.18	phosphoenolpyruvate carboxykinase	cytosol	metabolism, carbohydrate
NAN1	orf19.2688	1.18	Putative U3 snoRNP protein	nucleus	ribosome biogenesis, rRNA processing
NOG1	CAALFM_C306030WA	1.17	Putative GTPase	cytosol	ribosome biogenesis, rRNA processing

PES1	orf19.4093	1.17	Pescadillo homolog	nucleus	ribosome biogenesis, rRNA processing
MPP10	orf19.1915	1.17	Putative SSU processome and 90S preribosome component	nucleus	ribosome biogenesis, rRNA processing
hypothetical	orf19.5847	1.16	RNA polymerase III activity	nucleus	transcription
RPL7	orf19.3867	1.16	Ribosomal protein L7	nucleus	ribosome biogenesis, rRNA processing
YTM1	orf19.4815	1.16	biogenesis of the large ribosomal subun	nucleus	ribosome biogenesis, rRNA processing
KAR4	orf19.3736	1.16	Transcription factor; required for gene regulation in response to pheromones	nucleus	transcription
PWP2	orf19.3276	1.15	Putative 90S pre-ribosomal component	nucleus	ribosome biogenesis, rRNA processing
RBE1	orf19.7218	1.15	Pry family cell wall protein; Rim101	cell wall	cell wall biogenesis
YHM1	orf19.685	1.14	mitochondrial carrier protein	mitochondrion, plasma membrane	cellular iron ion homeostasis
NA	orf19.6090	1.14	Putative nucleolar protein	nucleus	ribosome biogenesis, rRNA processing
DRS1	orf19.7635	1.14	Putative nucleolar DEAD-box protein	nucleus	ribosome biogenesis, rRNA processing
YmL22	orf19.3367	1.13	mitochondrial large ribosomal subunit component	nucleus	ribosome biogenesis, rRNA processing
SIK1	orf19.7569	1.13	U3 snoRNP protein	nucleus	ribosome biogenesis, rRNA processing
hypothetical	(orf19.5038)	1.12	Predicted tRNA (guanine) methyltransferase activity		

BUD23	orf19.1966	1.12	Putative methyltransferase	nucleus	ribosome biogenesis, rRNA processing
RPF2	orf19.3553	1.11	pre-rRNA processing protein	nucleus	ribosome biogenesis, rRNA processing
hypothetical	orf19.3463	1.11	Putative GTPase;	nucleus	ribosome biogenesis, rRNA processing
RIX7	orf19.4219	1.11	Putative ATPase of the AAA family	nucleus	ribosome biogenesis, rRNA processing
hypothetical	orf19.3184	1.11	unknown, predicted role in vesicle-mediated transport	unknown	unknown
hypothetical	orf19.1772	1.11	unknown	unknown	unknown
NOP13	orf19.6766	1.11	Ortholog of S. cerevisiae Nop13; a nucleolar protein found in preribosomal complexes	nucleus	ribosome biogenesis, rRNA processing
C1_03790C_A	orf19.1030	1.10	Putative peptidyl-prolyl cis-trans isomerase	nucleus	ribosome biogenesis, rRNA processing
CR_04160C_A	orf19.500	1.10	Ortholog(s) have tRNA (adenine-N1-)-methyltransferase	nucleus	tRNA processing
hypothetical	orf19.1772	1.09	unknown	unknown	unknown
RRP6	orf19.58	1.09	Putative nuclear exosome exonuclease component	nucleus	ribosome biogenesis, rRNA processing
CR_00570W_A	orf19.7478	1.09	Putative enzyme with di-trans, poly-cis-decaprenylcistransferase activity	mitochondrion	farnesyl diphosphate biosynthetic process
MRT4	orf19.5550	1.08	Putative mRNA turnover protein	nucleus	ribosome biogenesis, rRNA processing
hypothetical	orf19.7664	1.08	unknown	unknown	unknown
hypothetical	orf19.5279	1.08	Orthologs have structural constituent of ribosome activity and role in cellular respiration, regulation of mitochondrial DNA metabolic process.	mitochondrion	mitochondrial electron transport and stability

MRPS17	orf19.4176	1.08	Orthologs have structural constituent of ribosome activity and mitochondrial small ribosomal subunit localization	mitochondrion	mitochondrial electron transport and stability
hypothetical	orf19.1772	1.08	unknown	unknown	unknown
C7_04140C_A	orf19.7160	1.08	unfolded protein binding	nucleus	oxidative stress response
Mrpl4	orf19.6136	1.08	a mitochondrial ribosomal protein of the large subunit	mitochondrion	mitochondrial protein translation
PWP1	orf19.4176	1.07	a mitochondrial ribosomal protein of the small subunit	mitochondrion	mitochondrial protein translation
GAR1	orf19.1164	1.07	Putative H/ACA snoRNP pseudouridylase complex protein	nucleus	ribosome biogenesis, rRNA processing
FRE10	orf19.1415	1.07	Major cell-surface ferric reductase under low-iron conditions	plasma membrane, cell surface	iron transport
NA	orf19.7107	1.07	unknown, Ortholog(s) have role in ribosomal large subunit biogenesis and cytoplasm, nucleus localization	nucleus	ribosome biogenesis, rRNA processing
GUA1	orf19.4813	1.07	GMP synthase	nucleus	ribosome biogenesis, rRNA processing
Mrps35	orf19.3559	1.07	Mrps35p is a structural constituent of ribosome	nucleus	ribosome biogenesis, rRNA processing
ERB1	orf19.1047	1.07	large ribosomal subunit rRNA binding	nucleus	ribosome biogenesis, rRNA processing
C4_05260W_A	orf19.2631	1.06	Subunit of Elongator complex	nucleus, cytoplasm	ribosome biogenesis, rRNA processing
RSM7	orf19.4018	1.06	ribosome activity and mitochondrial small ribosomal subunit localization	mitochondrion	mitochondrial protein translation
hypothetical	orf19.402	1.06	ribosome activity and mitochondrial small ribosomal subunit localization	cytosol	ribosome biogenesis, rRNA processing

MAK21	orf19.591	1.05	large ribosomal subunit rRNA binding	nucleus	ribosome biogenesis, rRNA processing
UTP8	orf19.5436	1.05	Nucleolar protein	nucleus	ribosome biogenesis, rRNA processing
NOP1	orf19.3138	1.05	Nucleolar protein	nucleus	ribosome biogenesis, rRNA processing
YOR1	orf19.1783	1.05	ABC-type plasma membrane transporter	plasma membrane, cell surface	transport
UTP22	orf19.1569	1.05	Putative U3 snoRNP protein	nucleus	ribosome biogenesis, rRNA processing
hypothetical	orf19.7011	1.04	putative protein with role in maturation of SSU-rRNA	nucleus	ribosome biogenesis, rRNA processing
RPC31	orf19.2831	1.04	Putative RNA polymerase III subunit C31;	RNA polymerase III complex	transcription
hypothetical	orf19.1578	1.04	Rrp5, an RNA binding protein	nucleus	ribosome biogenesis, rRNA processing
NOP2	orf19.501	1.04	RNA binding protein	nucleus	ribosome biogenesis, rRNA processing
hypothetical	orf19.1697	1.04	Ortholog(s) have role in cytoplasmic translation, poly(A)+ mRNA export from nucleus and cytoplasm localization	cytoplasm	translation
ELF1	orf19.7332	1.04	Putative mRNA export protein	plasma membrane, cytoplasm	ribosome biogenesis, rRNA processing
hypothetical	orf19.2299	1.04	protein tag	cytoplasm	stress response
YmL24/YmL14	orf19.828	1.04	ribosomal large subunit protein	mitochondrion	translation
UTP9	orf19.6710	1.04	Small subunit processome protein	nucleus	ribosome biogenesis, rRNA processing

NRI1	orf19.1441	1.03	Component of the RSC chromatin remodeling complex	RSC-type complex	unknown
MRPS9	orf19.5230	1.03	Mitochondrial ribosomal protein S9	mitochondrion	translation
HGH1	orf19.4587	1.03	Putative HMG1/2-related protein	unknown	protein folding
hypothetical	orf19.1662	1.03	unknown	mitochondrion	ribosome biogenesis, rRNA processing
hypothetical	orf19.4532	1.03	unknown	unknown	unknown
hypothetical	orf19.439	1.03	DNA binding, DNA strand exchange activity, single-stranded DNA binding, structural constituent of ribosome activity	mitochondrion	ribosome biogenesis, rRNA processing
TRM1	orf19.3265	1.02	Putative N2,N2-dimethylguanine tRNA methyltransferase	mitochondrion	ribosome biogenesis, rRNA processing
hypothetical	orf19.5229	1.02	3'-5'-exoribonuclease	nucleus	ribosome biogenesis, rRNA processing
MIS12	orf19.7534	1.02	Mitochondrial C1-tetrahydrofolate synthase precursor	unknown	unknown
MRPL37	orf19.755	1.02	Putative mitochondrial large subunit ribosomal protein	mitochondrion	ribosome biogenesis, rRNA processing
hypothetical	orf19.1565	1.02	unknown	unknown	unknown
RPA190	orf19.1839	1.02	Putative RNA polymerase I subunit A190	nucleus	transcription
NA	orf19.5698	1.01	Putative mitochondrial ribosomal protein of the large subunit	mitochondrial large ribosomal subunit	translation
ZPR1	orf19.3300	1.01	Protein with putative zinc finger	cytoplasm, nucleus	transcription
YNK1	orf19.4311	1.01	Nucleoside diphosphate kinase	cytoplasm, cell wall	transcription, DNA replication, stress
hypothetical	orf19.4204	1.01	unknown	unknown	unknown
FCA1	orf19.4195.1	1.01	Cytosine deaminase	biofilm	metobolism - cytosine
hypothetical	orf19.1662	1.01	a mitochondrial ribosomal protein of the small subunit	mitochondrion	mitochondrial protein translation

PHO84	orf19.655	1.01	High-affinity phosphate transporter	plasma membrane	transport - phosphate
hypothetical	orf19.863	1.01	a mitochondrial ribosomal protein of the large subunit	mitochondrion	mitochondrial protein translation
hypothetical	orf19.5168	1.00	unknown	unknown	unknown
hypothetical	orf19.512	1.00	Ortholog of S. cerevisiae Kre33	preribosome, small subunit precursor	ribosome biogenesis, rRNA processing
hypothetical	orf19.1662	1.00	a mitochondrial ribosomal protein of the small subunit	mitochondrion	mitochondrial protein translation
hypothetical	orf19.6853	1.00	unknown	unknown	unknown
RPO41	orf19.6041	1.00	Putative mitochondrial RNA polymerase	mitochondrion	DNA replication
hypothetical	orf19.2019	1.00	putative component of the mitochondrial ribosome	mitochondrial large ribosomal subunit	mitochondrial protein translation
MRPL40	orf19.484	0.99	putative component of the mitochondrial ribosome	mitochondrial large ribosomal subunit	mitochondrial protein translation
HPT1	orf19.5832	0.99	Putative hypoxanthine-guanine phosphoribosyltransferase	biofilm	metabolism - GMP and IMP salvage
hypothetical	orf19.2489	0.99	Putative karyopherin beta	nucleus, cytoplasm	nuclear import
hypothetical	orf19.2259	0.99	a mitochondrial ribosomal protein of the large subunit	mitochondrion	mitochondrial protein translation
TIM9	orf19.6696	0.99	Predicted protein of the mitochondrial intermembrane space	TIM22 mitochondrial import inner membrane insertion complex	mitochondrail protein transport
IDH1	orf19.4826	0.99	mitochondrial Isocitrate dehydrogenase	mitochondria	metabolism - TCA cycle
MRP7	orf19.7203	0.98	a mitochondrial ribosomal protein of the large subunit	mitochondrion	mitochondrial protein translation
IDH2	orf19.5791	0.98	mitochondrial Isocitrate dehydrogenase	mitochondria	metabolism - TCA cycle
CAALFM_CR01370CA	orf19.2520	0.98	a mitochondrial ribosomal protein of the small subunit	mitochondrion	mitochondrial protein translation

Supplemental Table 7: Genes common to two or more conditions

Comparison (number)	Shared Genes – orf #/name
Downregulated in Mechanical Shear and NSS (3)	orf19.1691/hypothetical, orf19.4945/MSH6, orf19.3051/hypothetical
Upregulated in Mechanical Shear and NSS (6)	orf19.5741/ALS1, orf19.6090/hypothetical, orf19.3367/ Mitochondrial 54S ribosomal protein YmL22, orf19.4697/MDN1, orf19.7624/BFR2, orf19.1833/CBF5, orf19.7215/hypothetical
Downregulated in PEG and NSS (13)	
Upregulated between PEG and NSS (10)	orf19.7675/Yml25, orf19.7514/PCK1, orf19.6854/YHM1, orf19.5832/HPT1, orf19.828/Yml24/Yml14, orf19.5791/IDH2, orf19.4311/YNK1, orf19.3797/Yml11, orf19.6696/TIM9, orf19.2520/ CAALFM_CR01370CA
Upregulated between Mechanical and PEG (89)	orf19.5049/hypothetical, orf19.3893/SCW11, orf19.5500/MAK16, orf19.7160/hypothetical, orf19.3630/RRP8, orf19.11240/CHR1, orf19.4273/hypothetical, orf19.2386/ CAALFM_CR03360WA, orf19.2934/hypothetical, orf19.1708/RRT14, orf19.59/REI1, orf19.76/SPB1, orf19.563/RRP15, orf19.6236/NOP6, orf19.2090/hypothetical, orf19.2758/PGA38, orf19.5299/ECM1, orf19.2998/TSR2, orf19.813/hypothetical, orf19.2287/RPA12, orf19.7586/CHT3, orf19.5267/hypothetical, orf19.2319/hypothetical, orf19.2167/hypothetical, orf19.5959/NOP14, orf19.107/hypothetical, orf19.2717/SAS10, orf19.6862/RIX1, orf19.2314/CGR1, orf19.6686/ENP2, orf19.3978/ CAALFM_C504910WA, orf19.1388/Nop16, orf19.414/RSM22, orf19.6955/HBR3, orf19.7050/NOP15, orf19.6886/hypothetical, orf19.3393/DBP9, orf19.661/KRR1, orf19.4933/FAD3, orf19.962/hypothetical, orf19.3287/BUD22, orf19.6297/hypothetical, orf19.1642/LOC1, orf19.809/NOP12, orf19.3470/hypothetical, orf19.4479/NOP9, orf19.1633/UTP4, orf19.4793/hypothetical, orf19.6014/RRS1, orf19.4191/RLP24, orf19.5850/NOC2, orf19.603/IMP4, orf19.5106/DIP2, orf19.5232/CSI2, orf19.6355/hypothetical, orf19.3831/PXR1, orf19.3477/PUS1, orf19.1791/hypothetical, orf19.1646/MRD1, orf19.5407/SOF1, orf19.7422/hypothetical, orf19.3724/ CAALFM_CR02420WA, orf19.4896/RPA34, orf19.5991/ DBP10, orf19.1915/MPP10, orf19.5847/ CAALFM_CR05550CA, orf19.3867/RPL7, orf19.3276/PWP2, orf19.7218/RBE1, orf19.7635/DRS1, orf19.7569/SIK1, orf19.3553/RPF2, orf19.3463/hypothetical, orf19.1772/hypothetical, orf19.1030/ FPR3 – downregulated in NSS, orf19.501/NOP2, orf19.1697/hypothetical, orf19.7332/ELF1, orf19.1441/NRI1, orf19.4532/hypothetical, orf19.3265/TRM1, orf19.7534/MIS12, orf19.1565/DUS3, orf19.1839/RPA190, orf19.3300/ZPR1, orf19.1569/UTP22, orf19.7160/hypothetical, orf19.1164/GAR1, orf19.512/NAT10
Downregulated between Mechanical and PEG (14)	orf19.822/HSP21, orf19.449/hypothetical, orf19.2803/HEM13 orf19.4980/SSA or HSP70, orf19.7676/XYL2, orf19.3302/hypothetical

	orf19.1785/hypothetical, orf19.3051/hypothetical, orf19.5813/hypothetical C2_02920W_A , orf19.882/HSP78, orf19.6387/HSP104, orf19.411/hypothetical, orf19.489/DAP1, orf19.2107.1/STF2
Downregulated among all three conditions (4)	orf19.2947/SNZ1, orf19.33/hypothetical, orf19.689/PLB1, orf19.2356/CRZ2
Upregulated among all three conditions (3)	orf19.6090/hypothetical, orf19.4176/MRPS17, orf19.3367/Yml22

, ,
